# Novel Metal-Free Synthesis of 3-Substituted Isocoumarins and Evaluation of Their Fluorescence Properties for Potential Applications

**DOI:** 10.3390/molecules29112449

**Published:** 2024-05-23

**Authors:** Mei Sun, Chong-Yang Zeng, Lu-Lu Bu, Mai Xu, Kai Chen, Jia-Lin Liu, Tao Zhang, Jia-You Dai, Jia-Xin Hong, Ming-Wu Ding

**Affiliations:** 1 School of Chemistry and Materials Engineering, Huainan Normal University, Huainan 232038, China; hssm202106@163.com (M.S.); cyzeng1028@126.com (C.-Y.Z.); lulubuvip@163.com (L.-L.B.); xumai2215@163.com (M.X.); kaichen8488@163.com (K.C.); ljl20048174@163.com (J.-L.L.); zt20040617@163.com (T.Z.); djy04514@163.com (J.-Y.D.); 2 Key Laboratory of Pesticide & Chemical Biology of Ministry of Education, Hubei International Scientific and Technological Cooperation Base of Pesticide and Green Synthesis, Central China Normal University, Wuhan 430079, China; 3 College of Food Science and Engineering, Jiangxi Agricultural University, Nanchang 330045, China

**Keywords:** isocoumarin, Wittig reaction, metal-free, phosphonium salt, fluorescence property

## Abstract

A novel metal-free synthesis of 3-substituted isocoumarins through a sequential O-acylation/Wittig reaction has been established. The readily accessible (2-carboxybenzyl)-triphenylphosphonium bromide and diverse chlorides produced various 1*H*-isochromen-1-one in the presence of triethylamine, employing sequential O-acylation and an intramolecular Wittig reaction of acid anhydride. Reactions using these facile conditions have exhibited high functional group tolerance and excellent yields (up to 90%). Moreover, the fluorescence properties of isocoumarin derivatives were evaluated at the theoretical and experimental levels to determine their potential application in fluorescent materials. These derivatives have good photoluminescence in THF with a large Stokes shift and an absolute fluorescence quantum yield of up to 14%.

## 1. Introduction

**Scheme 1:**
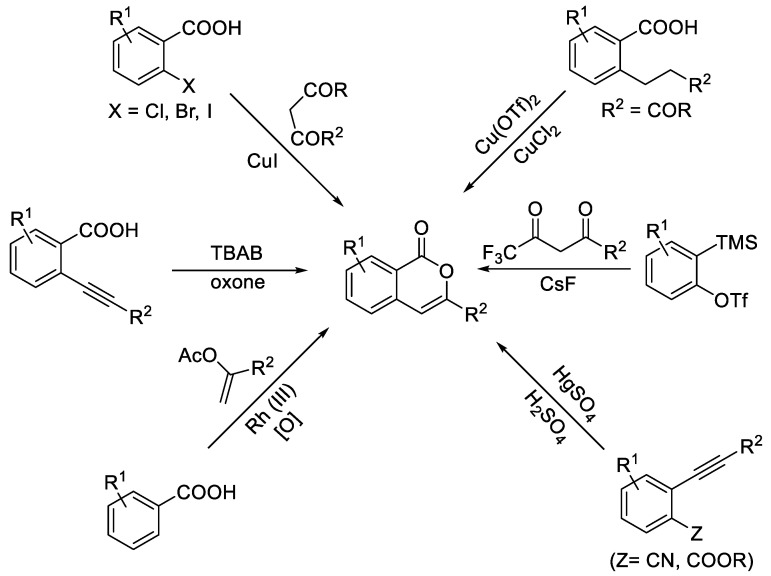
Synthesis of isocoumarins.

Isocoumarins, especially 3-substituted isocoumarins, constitute an important class of heterocyclic scaffolds owing to their diverse biological activities, including antibacterial [1], enzyme inhibition [2], antifungal [3], and antipheromonal effects [4], as well as anticancer [5] and anti-HIV properties [6]. Additionally, there are important applications in organic light-emitting materials [7,8,9,10,11,12,13]. Given their great importance, substantial efforts have been dedicated to developing new synthetic methods for the preparation of isocoumarins, driven by their potential applications (Scheme 1) [14]. Classical methods for synthesizing these 3-substituted isocoumarins were reported by Barry in 1964, Oliver in 1984, and Pal in 2011 [15,16,17]. These synthetic pathways involved multiple reactions, the use of toxic reagents, and harsh reaction conditions. Subsequently, the synthesis of these molecules gained popularity through metal-catalyzed cyclization (Scheme 1) [18,19,20,21]. For instance, 3-substituted isocoumarins were prepared either by Rh (III)-catalyzed oxidative coupling of benzoic acids with geminal substituted vinyl acetates [18] or by a CuI-catalyzed domino coupling/addition/deacylation process [19]. In addition, 3-substituted isocoumarins were recently found to be reported via sequential Rh(III)-catalyzed C–H activation/annulation [20]. These synthetic methodologies still necessitated the use of hazardous metallic reagents and multipart procedures or exhibited limited functional group tolerance. Furthermore, the same possible synthesis strategy for the construction of the isocoumarin nucleus has hardly been proposed under metal-free conditions (Scheme 1) [22,23]. Consequently, to overcome these limitations and to prepare various isocoumarins, a new metal-free and efficient synthetic method for variable isocoumarins is still desirable in synthetic organic and fluorescent molecule research.

The Wittig reaction is considered to be a powerful method for constructing carbon-carbon unsaturated bonds by converting the C=O double bond of an aldehyde or ketone to a C=C double bond [24,25,26]. Since its discovery more than half a century ago, the Wittig reaction has been widely utilized in organic synthesis due to its simple synthesis, excellent functional group tolerance, and environmental friendliness. Moreover, intramolecular Wittig reactions have been recognized as a valuable strategy for the synthesis of a variety of heterocycles [27,28], although a Wittig reaction involving a variety of carbonyl reagents (e.g., aldehydes, ketones, esters, and amides) has found widespread application in the synthesis of structurally diverse compounds [29,30,31,32]. Intermolecular Wittig reactions incorporating acid anhydrides as carbonyl components have been less commonly reported [33,34]. Continuing our exploration of heterocycle synthesis through Wittig reactions [35,36,37,38,39,40], we propose a novel, metal-free, and efficient method for the synthesis of 3-substituted isocoumarins by O-acylation/Wittig reaction, utilizing readily available (2-carboxybenzyl)triphenylphosphonium bromide **3** as the starting material. Finally, we evaluate the fluorescence properties of isocoumarin derivative **4** at both theoretical and experimental levels to assess its potential application in fluorescent materials.

## 2. Results and Discussion

**Scheme 2:**
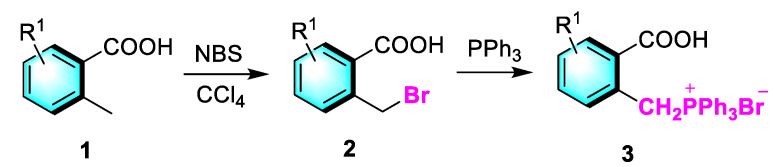
Synthesis of of (2-carboxybenzyl)triphenylphosphonium bromide **3**.

The readily accessible starting material (2-carboxybenzyl)triphenylphosphonium bromide **3** was synthesized as depicted in Scheme 2 [35]. In this process, the reaction between 2-methylbenzoic acid **1** and *N*-Bromosuccinimide (NBS) in CCl_4_ took place at reflux temperature over a duration of 4–8 h, yielding 2-(bromomethyl)benzoic acid **2** in 80% yield. Next, the 2-(bromomethyl)benzoic acid **2** reacted with triphenylphosphine in acetone at room temperature, producing (2-carboxybenzyl)triphenylphosphonium bromide **3** in 90% yield.

In order to optimize the conditions for the O-acylation–Wittig reaction sequence, (2-carboxybenzyl)triphenylphosphonium bromide **3a** and benzoyl chloride were selected as model substrates (Table 1). When the reaction was conducted in the presence of DMAP (2 equiv) in CH_2_Cl_2_ at room temperature for 12 h, the expected 3-phenyl-1H-isochromen-1-one **4a** was not obtained (Table 1, Entry 1). Subsequently, we screened different bases including NEt_3_, DBU, t-BuOK, K_2_CO_3_, and NaOH (Table 1, Entries 2–6), and the reaction gave comparably better yields of **4a** using Et_3_N (Entry 2, 79%). The suitable basicity of NEt_3_ may influence both the Wittig reaction and the O-acylation reaction steps. Subsequently, NEt_3_ was employed as the base to investigate the impact of alternative solvents, including THF, CH_3_CN, DCE, 1,4-dioxane, and toluene, on the reaction (Table 1, Entries 7–11), the results indicated that 3-phenyl-1H-isochromen-1-one **4a** were obtained in the better-isolated yield (79%, Table 1, Entry 2). To examine the temperature-dependent effects on the reaction, toluene was employed as the solvent, and the reaction was conducted at 80 °C or 110 °C for 2–6 h, respectively (Table 1, Entries 12–13). The product was obtained with a higher yield of 82% (Table 1, Entry 13). This suggests that the reaction temperature influences both the reaction rate and the efficacy of the Wittig reaction. Thus, the optimum reaction condition for the preparation of 3-phenyl-1H-isochromen-1-one **4a** was found (Table 1, Entry 13).

Under optimal conditions, we initiated an investigation into the reaction’s scope, observing that the yields of product **4** were primarily influenced by the R^2^ group (Table 2). When R^2^ was an aromatic moiety (Table 2, compounds **4a**–**4t**), good yields (72–90%) were achieved with various electrono-accepting and electron-withdrawing groups (CH_3_, *t*-Bu, CH_3_O, 3,4,5-(CH_3_O)_3_, F, Cl, and Br) on the benzene ring. Notably, compounds were successfully synthesized even with a strong electron-withdrawing group (CF_3_) on the benzene ring. Furthermore, when R^2^ comprised an ortho-substituted phenyl group, compounds **4j** and **4s** were synthesized successfully (Table 2, 75% and 79%). Using Cinnamoyl chloride as an acyl chloride reactant, the preparation of 1H-isochromen-1-ones was also accomplished successfully (Table 2, **4h** and **4r**). It is noteworthy that R^2^ consisted of a thiophene ring or naphthalene ring, and both yielded satisfactorily (Table 2, 80-86%, compounds **4k**, **4q**, and **4t**). Moderate yields (60%) were obtained when R^2^ is an alkyl group, likely due to its relatively lower reactivity (Table 2, compounds **4u**). In summary, high functional group tolerance for this reaction was proved.

**Scheme 3:**
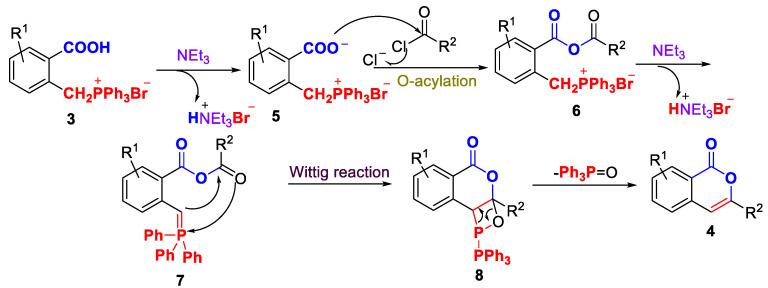
Proposed mechanism for the formation of **4**.

Based on the presented observation and previous literature reports [27,33,35], Scheme 3 depicts a plausible mechanism for the sequential O-acylation/Wittig process. In the presence of the base NEt_3_, phosphonium salt **3** is converted into conjugate base **5**, which undergoes O-acylation with an acyl chloride to form an anhydride **6**. This process releases one equivalent of HBr, which is captured by NEt_3_. Then, a second equivalent of triethylamine deprotonnates the anhydride **6** to phosphorus ylide **7**. This process releases one equivalent of HBr, which is also captured by NEt_3_. Subsequently, phosphorus ylide **7** undergoes an intramolecular Wittig reaction to form oxaphosphetane intermediate **8**, accompanied by [2+2] cycloaddition. Intermediate **8** then converts to 1H isochrom-1-one **4**, releasing equimolar amounts of oxygen and phosphorus.

Coumarin, a typical fluorophore, has been widely studied in dyes, pigments, fluorescent probes, and other fields due to its favorable optical properties. Isocoumarins are no exception, owing to their similar conjugated structures. Therefore, to further demonstrate the synthetic utility of our approach, isocoumarin derivatives **4** with different structures were screened to assess their optical properties. Considering the conjugated structure and substituent types of these derivatives, **4a**, **4h**, **4j**, **4k**, **4p**, and **4u** were specially selected as a representative for further investigation (Table 3).

**Figure 1:**
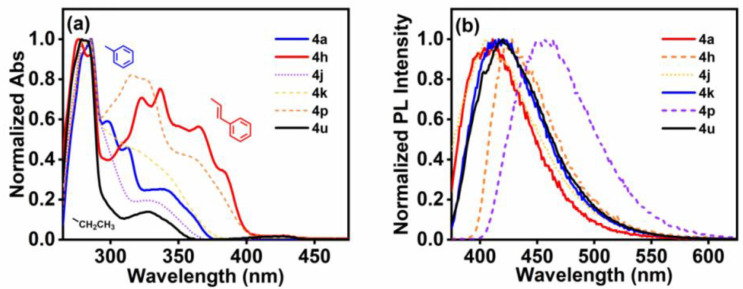
UV-vis absorption spectra (**a**) and fluorescence emission spectra (**b**) of isocoumarin derivatives **4** in THF (*c* = 10^−5^ mol/L).

The isocoumarin derivatives exhibit very similar absorption spectra in THF (Figure 1a), with a maximum absorption peak (λ_a_) of about around 280 nm and one or multiple bands in the range of 300–400 nm. The molar extinction coefficients (*ε*) of these absorption peaks are greater than 10^4^ L·mol^−1^·cm^−1^, confirming that intramolecular electron transitions are permitted [41]. The absorption at shorter wavelengths mainly arises from π-π* transition absorption, while the absorption at longer wavelengths is primarily attributed to *n*-π* transition absorption and intramolecular charge transfer absorption generated by the donor–acceptor (D-A) system [42]. The long-wavelength, lower-energy band absorption is attributed to the transition of the dominant H (HOMO) → L (LUMO), while the maximum absorption peak is mainly attributed to the H → L+1 or H-2 → L+1 transition. The difference in absorption intensity is related to the various oscillator strengths (*f_osc_*). Like most molecules, derivatives **4** with larger conjugated structures have larger and broader UV absorption spectra, with **4h** > **4a** > **4u**. Furthermore, fluorescence emission spectra were used to roughly assess the photoluminescence behavior of these compounds (Figure 1b). Their emission wavelengths are primarily concentrated around 420 nm, resulting in purple emission. Notably, the emission wavelength of **4p** is 457 nm with blue fluorescence. Their Stokes shift values range from 3978 to 6678 cm^−1^. This value is comparable to that of most small-molecule fluorescent compounds at this stage [43]. This may facilitate the material’s ability to overcome self-absorption and be effectively applied to probes or imaging. Of course, subsequent studies can also prepare isocoumarin derivatives with larger Stokes shifts by molecular design (i.e., introducing electron-withdrawing groups, extending conjugated systems, etc.) [44]. In THF, the absolute fluorescence quantum yields of **4h** can reach 14%, while **4u**, with the smallest conjugated structure, exhibits a hardly detectable fluorescence quantum yield.

**Figure 2:**
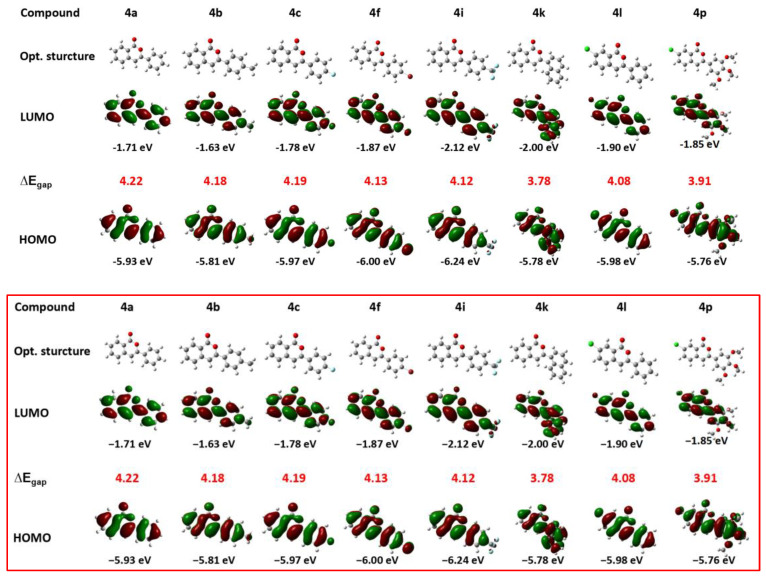
Optimized conformation, frontier molecular orbital electron cloud distribution, energy level and band gap of isocoumarin derivatives **4**.

Additionally, the photophysical properties of isocoumarin derivatives **4** were evaluated at the theoretical level (Figure 2). Structure optimization, frequency calculation, and calculation of the electron cloud distribution of frontier molecular orbitals for isocoumarin derivatives **4** were conducted using Gaussian 09 with the theoretical level of B3LYP, 6-31g (d). From the perspective of optimized structure, almost all molecules maintain good planarity, except for the naphthalene-substituted **4k**. The large volume of naphthalene unavoidably leads to distorted dihedral angles. Despite this, the relatively planar structures ensure a certain rigidity, which is conducive to fluorescence emission. The Highest Occupied Molecular Orbital (HOMO) electron cloud of these derivatives is predominantly distributed on the conjugated benzene skeleton, while the Lowest Unoccupied Molecular Orbital (LUMO) exhibits an intramolecular charge transfer (ICT) effect due to the influence of the ester group and electron-withdrawing substitutions (such as -Cl, -CF_3_, etc.). However, this intramolecular charge transfer (ICT) is relatively weak due to the small size of the structure and the limited separation between electron-withdrawing and electron-donating groups. Therefore, if we aim to modulate the rich fluorescence behavior through ICT (i.e., stimulus-responsive luminescence, etc.), these aspects must be improved upon. From the perspective of the transition band gap, electron-withdrawing groups can effectively reduce the band gap level more, whether they are located on the side group benzene (compared **4b** and **4i**) or on the fused benzene (compared **4a** and **4l**). In addition, a larger conjugated structure, compared to compounds **4a** with **4k**, also significantly reduces the band gap. Indeed, a low band gap is beneficial for electron transition and the improvement of semiconductor properties [45]. In summary, these theoretical data provide the necessary theoretical basis for the subsequent design and development of fluorescent materials derived from isocoumarin.

## 3. Materials and Methods

### 3.1. General Information

Melting points were determined using an X-4 model apparatus and were uncorrected. ^1^H NMR were recorded in CDCl_3_ on a Varian Mercury 600 spectrometer and resonances were relative to TMS. ^13^C NMR spectra were recorded in CDCl_3_ on a Varian Mercury 600 (150 MHz) with complete proton decoupling spectrophotometers (CDCl_3_: 77.0 ppm). HRMS was measured on an Agilent 6224 TOF LC/MS spectrometer. UV-vis absorption spectra were measured on a TU-1901 spectrophotometer. Fluorescence spectra were measured on the F97pro fluorescence spectrometer and HORIBA Fluoromax-4p. Density functional theory (DFT) calculations were conducted to determine the optimized conformation, oscillator strength (*f_osc_*), and UV absorption properties at Gaussian 09. The optimized conformation and frequency calculations were performed at the B3LYP/6-31g (d) theoretical level, while the excited state properties were investigated using time-dependent density functional theory (TDDFT) at the B3LYP/6-31g (d) level.

### 3.2. General Procedure for the Synthesis of 1H-isochromen-1-ones **4**

Anhydrous toluene (6 mL), NEt_3_ (0.28 mL, 2.0 mmol), and (2-carboxybenzyl)triphenylphosphonium bromide **3** (1 mmol) were initially added into a dry reaction flask. Subsequently, acyl chloride (1.1 mmol) in anhydrous toluene (2 mL) was added dropwise over a time period of approximately 30 min. The mixture was maintained at 110 °C for 2–4 h, with the reaction progress monitored by TLC. The solvent was then evaporated under reduced pressure, and the residue was purified by flash chromatography on silica gel (petroleum ether/ethyl acetate = 60:1–15:1, *V/V*) to yield compound **4**.

*3-phenyl-1H-isochromen-1-one (**4a**)* [19]. (Rf = 0.65, petroleum ether/ethyl acetate = 10:1, *V/V*), white solid (yield 0.182 g, 82%), mp 84–85 °C; ^1^H NMR (CDCl_3,_ 600 MHz) δ (ppm) 8.28 (d, *J* = 6.8 Hz, 1H), 7.86 (d, *J* = 6.0 Hz, 2H), 7.69 (s, 1H), 7.51–7.39 (m, 5H), 6.92 (s, 1H).; ^13^C NMR (CDCl_3,_ 150 MHz) δ (ppm) 162.27, 153.43, 137.41, 134.84, 131.80, 129.91, 129.51, 128.77, 128.09, 125.97, 125.13, 120.38, 101.77. HRMS (ESI-TOF) *m/z* [M+H]^+^ Calcd for C_15_H_11_O_2_ 223.0754; found 223.0754.

*3-(p-tolyl)-1H-isochromen-1-one (**4b**)* [18]. (Rf = 0.66, petroleum ether/ethyl acetate = 10:1, *V/V*), white solid (yield 0.198 g, 84%), mp 88–89 °C; ^1^H NMR (CDCl_3,_ 600 MHz) δ (ppm) 8.28 (d, *J* = 7.8 Hz, 1H), 7.76 (d, *J* = 8.0 Hz, 2H), 7.69 (t, *J* = 7.5 Hz, 1H), 7.48–7.43 (m, 2H), 7.25 (d, *J* = 7.8 Hz, 2H), 6.88 (s, 1H), 2.39 (s, 3H); ^13^C NMR (CDCl_3,_ 150 MHz) δ (ppm) δ 162.27, 153.57, 140.07, 137.50, 134.65, 129.39, 129.35, 128.92, 127.71, 125.68, 124.95, 120.15, 100.89, 21.23. HRMS (ESI-TOF) *m/z* [M+H]^+^ Calcd for C_16_H_13_O_2_ 237.0910; found 237.0909.

*3-(4-fluorophenyl)-1H-isochromen-1-one (**4c**)* [46]. (Rf = 0.64, petroleum ether/ethyl acetate = 10:1, *V/V*), white solid (yield 0.216g, 90%), mp 105–106 °C; ^1^H NMR (CDCl_3,_ 600 MHz) δ (ppm) 8.18 (s, 1H), 7.75 (s, 2H), 7.61 (s, 1H), 7.38 (s, 2H), 7.03 (s, 2H), 6.77 (s, 1H); ^13^C NMR (CDCl_3,_ 150 MHz) δ (ppm) 164.34, 162.68, 161.97, 152.45, 137.19, 134.78, 129.43, 128.02, 127.07, 127.02, 125.77, 120.10, 115.84, 115.69, 101.38. HRMS (ESI-TOF) *m/z* [M+H]^+^ Calcd for C_15_H_10_FO_2_ 241.0659; found 241.0646.

*3-(4-methoxyphenyl)-1H-isochromen-1-one (**4d**)* [18]. (Rf = 0.61, petroleum ether/ethyl acetate = 8:1, *V/V*), white solid (yield 0.209 g, 83%), mp 128–129 °C; ^1^H NMR (CDCl_3,_ 600 MHz) δ (ppm) 8.26 (d, *J* = 7.0 Hz, 1H), 7.79 (d, *J* = 7.3 Hz, 2H), 7.67 (s, 1H), 7.43 (s, 2H), 6.95 (d, *J* = 7.4 Hz, 2H), 6.80 (s, 1H), 3.84 (s, 3H); ^13^C NMR (CDCl_3,_ 150 MHz) δ (ppm) 164.76, 163.74, 162.94, 162.44, 161.49, 161.01, 153.63, 137.84, 134.77, 129.54, 127.60, 126.74, 125.66, 124.45, 120.06, 114.18, 100.18, 55.36. HRMS (ESI-TOF) *m/z* [M+H]^+^ Calcd for C_16_H_13_O_3_ 253.0859; found 253.0859.

*3-(m-tolyl)-1H-isochromen-1-one (**4e**)* [46]. (Rf = 0.67, petroleum ether/ethyl acetate = 10:1, *V/V*), white solid (yield 0.193 g, 82%), mp 100-101 °C; ^1^H NMR (CDCl_3,_ 600 MHz) δ (ppm) 8.28 (d, *J* = 8.0 Hz, 1H), 7.68 (d, *J* = 8.3 Hz, 2H), 7.65 (d, *J* = 7.7 Hz, 1H), 7.46 (dd, *J* = 7.2, 4.3 Hz, 2H), 7.32 (t, *J* = 7.6 Hz, 1H), 7.22 (d, *J* = 7.4 Hz, 1H), 6.91 (s, 1H), 2.40 (s, 3H); ^13^C NMR (CDCl_3,_ 150 MHz) δ (ppm) 162.37, 153.72, 140.04, 138.54, 137.55, 134.81, 131.80, 130.73, 129.57, 128.66, 128.01, 125.89, 125.81, 122.33, 120.43, 101.66, 21.42. HRMS (ESI-TOF) *m/z* [M+H]^+^ Calcd for C_16_H_13_O_2_ 237.0910; found 237.0910.

*3-(4-bromophenyl)-1H-isochromen-1-one (**4f**)* [47]. (Rf = 0.64, petroleum ether/ethyl acetate = 7:1, *V/V*), white solid (yield 0.267 g, 89%); mp 158–159 °C; ^1^H NMR (CDCl_3,_ 600 MHz) δ (ppm) 8.29 (d, *J* = 7.8 Hz, 1H), 7.72 (t, *J* = 9.3 Hz, 3H), 7.58 (d, *J* = 8.3 Hz, 2H), 7.50 (dd, *J* = 14.6, 7.6 Hz, 2H), 6.93 (s, 1H); ^13^C NMR (CDCl_3,_ 150 MHz) δ (ppm) 161.96, 152.50, 137.17, 134.95, 132.01, 130.82, 129.68, 128.40, 126.63, 126.02, 124.28, 120.74, 120.52, 102.08. HRMS (ESI-TOF) *m/z* [M+H]^+^ Calcd for C_15_H_10_BrO_2_ 300.9859; found 300.9859.

*3-(4-(tert-butyl)phenyl)-1H-isochromen-1-one (**4g**)* [47]. (Rf = 0.72, petroleum ether/ethyl acetate = 10:1, *V/V*), white solid (yield 0.217 g, 78%), mp 80–81 °C; ^1^H NMR (CDCl_3,_ 600 MHz) δ (ppm) 8.30 (d, *J* = 7.7 Hz, 1H), 7.82 (d, *J* = 8.3 Hz, 2H), 7.70 (t, *J* = 7.3 Hz, 1H), 7.48 (dd, *J* = 14.6, 7.9 Hz, 4H), 6.92 (s, 1H), 1.37 (s, 9H); ^13^C NMR (CDCl_3,_ 150 MHz) δ (ppm) 162.24, 153.56, 153.20, 137.50, 134.64, 129.39, 128.92, 127.71, 125.72, 125.61, 124.84, 120.19, 101.00, 31.02. HRMS (ESI-TOF) *m/z* [M+H]^+^ Calcd for C_19_H_19_O_2_ 279.1380; found 279.1379.

*(E)-3-styryl-1H-isochromen-1-one (**4h**).* (Rf = 0.63, petroleum ether/ethyl acetate = 10:1, *V/V*), white solid (yield 0.201 g, 81%); mp 116–117 °C; ^1^H NMR (CDCl_3,_ 600 MHz) δ (ppm) 8.24 (s, 1H), 7.64 (s, 1H), 7.48 (d, *J* = 6.0 Hz, 2H), 7.38 (dd, *J* = 24.4, 13.5 Hz, 5H), 7.30 (s, 1H), 6.65 (d, *J* = 15.9 Hz, 1H), 6.41 (s, 1H); ^13^C NMR (CDCl_3,_ 150 MHz) δ (ppm) 161.90, 152.33, 137.33, 135.57, 134.66, 132.64, 129.56, 128.67, 127.87, 126.96, 125.63, 120.57, 119.20, 105.64. HRMS (ESI-TOF) *m/z* [M+H]^+^ Calcd for C_17_H_13_O_2_ 249.0910; found 249.0911.

*3-(4-(trifluoromethyl)phenyl)-1H-isochromen-1-one (**4i**)* [47]. (Rf = 0.58, petroleum ether/ethyl acetate = 6:1, *V/V*), white solid (yield 0.206 g, 72%), mp 214–215 °C; ^1^H NMR (CDCl_3,_ 600 MHz) δ (ppm) 8.29 (d, *J* = 7.6 Hz, 1H), 7.96 (d, *J* = 7.8 Hz, 2H), 7.73 (t, *J* = 7.3 Hz, 1H), 7.69 (d, *J* = 7.8 Hz, 2H), 7.51 (d, *J* = 7.6 Hz, 2H), 7.02 (s, 1H); ^13^C NMR (CDCl_3,_ 150 MHz) δ (ppm) 161.75, 151.87, 136.83, 135.17, 135.03, 131.60, 131.39, 129.71, 128.81, 126.25, 125.78, 125.37, 120.77, 103.37. HRMS (ESI-TOF) *m/z* [M+H]^+^ Calcd for C_16_H_10_F_3_O_2_ 291.0627; found 291.0631.

*3-(o-tolyl)-1H-isochromen-1-one (**4j**).* (Rf = 0.70, petroleum ether/ethyl acetate = 10:1, *V/V*), white solid (yield 0.186 g, 79%), mp 90–91 °C; ^1^H NMR (CDCl_3,_ 600 MHz) δ (ppm) 8.32 (d, *J* = 7.9 Hz, 1H), 7.72 (t, *J* = 7.5 Hz, 1H), 7.51 (t, *J* = 7.6 Hz, 2H), 7.47 (d, *J* = 7.8 Hz, 1H), 7.34 (t, *J* = 7.4 Hz, 1H), 7.27 (d, *J* = 8.1 Hz, 2H), 6.60 (s, 1H), 2.50 (s, 3H); ^13^C NMR (CDCl_3,_ 150 MHz) δ (ppm) 162.55, 155.52, 137.45, 136.72, 134.81, 132.72, 131.02, 129.76, 129.54, 129.14, 128.89, 128.20, 125.95, 125.81, 120.28, 105.89, 20.57. HRMS (ESI-TOF) *m/z* [M+H]^+^ Calcd for C_16_H_13_O_2_ 237.0910; found 237.0910.

*3-(naphthalen-1-yl)-1H-isochromen-1-one (**4k**)* [47]. (Rf = 0.65, petroleum ether/ethyl acetate = 10:1, *V/V*), white solid (yield 0.231 g, 85%), mp 148–149 °C; ^1^H NMR (CDCl_3,_ 600 MHz) δ (ppm) 8.37 (d, *J* = 7.7 Hz, 1H), 8.24 (d, *J* = 7.6 Hz, 1H), 7.92 (dd, *J* = 21.7, 7.4 Hz, 2H), 7.74 (d, *J* = 7.0 Hz, 2H), 7.53 (d, *J* = 17.2 Hz, 5H), 6.80 (s, 1H); ^13^C NMR (CDCl_3,_ 150 MHz) δ (ppm) 162.63, 154.70, 137.42, 134.91, 133.74, 130.73, 130.52, 129.65, 128.57, 128.40, 127.69, 127.08, 126.26, 125.89, 125.09, 125.02, 120.50, 120.29, 107.11, 103.85. HRMS (ESI-TOF) *m/z* [M+H]^+^ Calcd for C_19_H_13_O_2_ 273.0910; found 237.0916.

*7-chloro-3-phenyl-1H-isochromen-1-one (**4l**).* (Rf = 0.63, petroleum ether/ethyl acetate = 9:1, *V/V*), white solid (yield 0.220 g, 86%), mp 138–139 °C; ^1^H NMR (CDCl_3,_ 600 MHz) δ (ppm) 8.19 (s, 1H), 7.80 (d, J = 5.3 Hz, 2H), 7.61 (d, J = 8.1 Hz, 1H), 7.41 (s, 4H), 6.87 (s, 1H); ^13^C NMR (CDCl_3,_ 150 MHz) δ (ppm) 160.95, 153.67, 135.66, 135.04, 133.59, 131.27, 130.04, 128.84, 128.69, 127.29, 125.00, 121.35, 100.78. HRMS (ESI-TOF) *m/z* [M+H]^+^ Calcd for C_15_H_10_ClO_2_ 257.0364; found 257.0365.

*7-chloro-3-(p-tolyl)-1H-isochromen-1-one (**4m**).* (Rf = 0.64, petroleum ether/ethyl acetate = 9:1, *V/V*), white solid (yield 0.227 g, 84%), mp 205–206 °C; ^1^H NMR (CDCl_3,_ 600 MHz) δ (ppm) 8.16 (s, 1H), 7.66 (d, *J* = 7.6 Hz, 2H), 7.58 (d, *J* = 7.1 Hz, 1H), 7.36 (d, *J* = 8.2 Hz, 1H), 7.20 (d, *J* = 7.3 Hz, 2H), 6.80 (s, 1H), 2.36 (s, 3H); ^13^C NMR (CDCl_3,_ 150 MHz) δ (ppm) 161.03, 153.83, 140.37, 135.83, 134.93, 133.25, 129.37, 128.74, 128.42, 127.14, 124.87, 121.15, 99.98, 21.23. HRMS (ESI-TOF) *m/z* [M+H]^+^ Calcd for C_16_H_12_ClO_2_ 271.0520; found 271.0522.

*7-chloro-3-(4-methoxyphenyl)-1H-isochromen-1-one (**4n**)*. (Rf = 0.59, petroleum ether/ethyl acetate = 9:1, *V/V*), white solid (yield 0.235 g, 82%), mp 183–184 °C; ^1^H NMR (CDCl_3,_ 600 MHz) δ (ppm) 8.16 (s, 1H), 7.72 (d, *J* = 8.1 Hz, 2H), 7.57 (d, *J* = 7.1 Hz, 1H), 7.35 (d, *J* = 8.1 Hz, 1H), 6.90 (d, *J* = 8.1 Hz, 2H), 6.73 (s, 1H), 3.82 (s, 3H); ^13^C NMR (CDCl_3,_ 150 MHz) δ (ppm) 161.10, 160.99, 153.73, 136.04, 134.94, 132.97, 128.74, 127.00, 126.56, 123.76, 120.88, 114.04, 99.13, 55.21. HRMS (ESI-TOF) *m/z* [M+H]^+^ Calcd for C_16_H_12_ClO_3_ 287.0469; found 287.0468.

*3-(4-(tert-butyl)phenyl)-7-chloro-1H-isochromen-1-one (**4o**).* (Rf = 0.63, petroleum ether/ethyl acetate = 10:1, *V/V*), white solid (yield 0.253 g, 81%), mp 236–237 °C; ^1^H NMR (CDCl_3,_ 600 MHz) δ (ppm) 8.24 (s, 1H), 7.78 (d, *J* = 7.9 Hz, 2H), 7.62 (d, *J* = 8.3 Hz, 1H), 7.47 (d, *J* = 8.2 Hz, 2H), 7.42 (d, *J* = 8.3 Hz, 1H), 6.87 (s, 1H), 1.35 (s, 9H); ^13^C NMR (CDCl_3,_ 150 MHz) δ (ppm) 161.24, 161.00, 154.12, 153.71, 136.06, 135.13, 133.50, 129.03, 128.70, 127.31, 125.83, 125.01, 121.48, 100.30, 34.85, 31.12. HRMS (ESI-TOF) *m/z* [M+H]^+^ Calcd for C_19_H_18_ClO_2_ 313.0990; found 313.0991.

*7-chloro-3-(3,4,5-trimethoxyphenyl)-1H-isochromen-1-one (**4p**).* (Rf = 0.60, petroleum ether/ethyl acetate = 3:1, *V/V*), white solid (yield 0.277 g, 80%), mp 168–169 °C; ^1^H NMR (CDCl_3,_ 600 MHz) δ (ppm) 8.22 (s, 1H), 7.64 (d, *J* = 8.3 Hz, 1H), 7.43 (d, *J* = 8.3 Hz, 1H), 7.04 (s, 2H), 6.84 (s, 1H), 3.95 (s, 6H), 3.91 (s, 3H); ^13^C NMR (CDCl_3,_ 150 MHz) δ (ppm) ^13^C NMR 161.05, 153.65, 153.46, 139.96, 139.63, 135.83, 135.22, 133.64, 129.01, 127.28, 126.93, 121.30, 102.52, 100.64, 60.95, 56.29. HRMS (ESI-TOF) *m/z* [M+H]^+^ Calcd for C_18_H_16_ClO_5_ 347.0681; found 347.0681.

*7-chloro-3-(naphthalen-1-yl)-1H-isochromen-1-one (**4q**).* (Rf = 0.64, petroleum ether/ethyl acetate = 9:1, *V/V*), white solid (yield 0.263 g, 86%), mp 195–196 °C; ^1^H NMR (CDCl_3,_ 600 MHz) δ (ppm) 8.31 (s, 1H), 8.19 (d, *J* = 7.6 Hz, 1H), 7.92 (dd, *J* = 24.2, 7.9 Hz, 2H), 7.70 (dd, *J* = 23.4, 7.6 Hz, 2H), 7.52 (dd, *J* = 14.5, 6.9 Hz, 3H), 7.44 (d, *J* = 8.2 Hz, 1H), 6.77 (s, 1H); ^13^C NMR (CDCl_3,_ 150 MHz) δ (ppm) 161.45, 155.05, 135.77, 135.24, 134.05, 133.72, 130.74, 130.53, 130.34, 129.09, 128.63, 127.74, 127.39, 127.18, 126.33, 125.00, 124.92, 121.62, 106.31. HRMS (ESI-TOF) *m/z* [M+H]^+^ Calcd for C_19_H_12_ClO_2_ 307.0520; found 307.0520.

*(E)-7-chloro-3-styryl-1H-isochromen-1-one (**4r**).* (Rf = 0.65, petroleum ether/ethyl acetate = 9:1, *V/V*), light yellow solid (yield 0.228 g, 81%), mp 205–206 °C; ^1^H NMR (CDCl_3,_ 600 MHz) δ (ppm) 8.18 (d, *J* = 9.0 Hz, 1H), 7.51 (d, *J* = 7.4 Hz, 2H), 7.45 (d, *J* = 15.9 Hz, 1H), 7.38 (t, *J* = 8.3 Hz, 4H), 7.32 (t, *J* = 7.2 Hz, 1H), 6.67 (d, *J* = 15.9 Hz, 1H), 6.36 (s, 1H); ^13^C NMR (CDCl_3,_ 150 MHz) δ (ppm) 160.87, 152.83, 135.89, 135.56, 135.15, 133.68, 133.45, 129.26, 129.00, 128.84, 127.16, 121.87, 119.01, 104.80. HRMS (ESI-TOF) *m/z* [M+H]^+^ Calcd for C_17_H_11_ClO_2_ 283.0520; found 283.0519.

*7-chloro-3-(o-tolyl)-1H-isochromen-1-one (**4s**).* (Rf = 0.67, petroleum ether/ethyl acetate = 9:1, *V/V*), white solid (yield 0.204 g, 75%), mp 100–101 °C; ^1^H NMR (CDCl_3,_ 600 MHz) δ (ppm) 8.29 (s, 1H), 7.67 (d, *J* = 6.7 Hz, 1H), 7.49 (d, *J* = 7.5 Hz, 1H), 7.43 (d, *J* = 8.3 Hz, 1H), 7.35 (t, *J* = 7.4 Hz, 1H), 7.28 (d, *J* = 8.5 Hz, 2H), 6.59 (s, 1H), 2.49 (s, 3H); ^13^C NMR (CDCl_3,_ 150 MHz) δ (ppm) 161.53, 161.39, 155.92, 155.80, 136.73, 135.82, 135.15, 133.89, 132.36, 131.10, 129.97, 129.11, 129.04, 127.29, 126.01, 105.10, 20.74. HRMS (ESI-TOF) *m/z* [M+H]^+^ Calcd for C_16_H_14_ClO_2_ 273.0677; found 273.0676.

*6-chloro-3-(thiophen-2-yl)-1H-isochromen-1-one (**4t**).* (Rf = 0.58, petroleum ether/ethyl acetate = 8:1, *V/V*), white solid (yield 0.210 g, 80%), mp 188–189 °C; ^1^H NMR (CDCl_3,_ 600 MHz) δ (ppm) 8.17 (d, *J* = 8.1 Hz, 1H), 7.58 (s, 1H), 7.44-7.32 (m, 3H), 7.10 (s, 1H), 6.67 (s, 1H); ^13^C NMR (CDCl_3,_ 150 MHz) δ (ppm) 160.84, 150.64, 141.60, 138.80, 135.13, 131.40, 128.27, 128.20, 127.99, 126.69, 125.10, 118.46, 99.66. HRMS (ESI-TOF) *m/z* [M+H]^+^ Calcd for C_13_H_8_ClO_2_S 262.9928; found 262.9928.

*6-chloro-3-ethyl-1H-isochromen-1-one (**4u**).* (Rf = 0.69, petroleum ether/ethyl acetate = 25:1, *V/V*), white solid (yield 0.125 g, 60%), mp 77–78 °C; ^1^H NMR (CDCl_3,_ 600 MHz) δ (ppm) 8.16 (d, *J* = 8.3 Hz, 1H), 7.39 (d, *J* = 8.1 Hz, 1H), 7.34 (s, 1H), 6.19 (s, 1H), 2.62–2.49 (m, 2H), 1.28 (t, *J* = 7.3 Hz, 3H); ^13^C NMR (CDCl_3,_ 150 MHz) δ (ppm) 162.14, 160.94, 141.27, 138.95, 131.11, 127.97, 124.55, 118.35, 101.07, 26.68, 11.07. HRMS (ESI-TOF) *m/z* [M+H]^+^ Calcd for C_11_H_10_ClO_2_ 209.0364; found 209.0364.

## 4. Conclusions

In summary, we have introduced a novel approach for the synthesis of 3-substituted isocoumarins through a sequential O-acylation/Wittig reaction, utilizing the readily accessible (2-carboxybenzyl)triphenylphosphonium bromide. Our method holds considerable significance for the synthesis of diverse fluorescent 3-substituted isocoumarins under environmentally friendly metal-free conditions, demonstrating notable functional group tolerance and good yields. Furthermore, the photophysical properties of some isocoumarin derivatives **4** at the experimental and theoretical levels were investigated and proved that these derivatives have further research and development value in the field of fluorescent materials.

## Figures and Tables

**Table 1:** Optimization of reaction conditions ^a,b^.

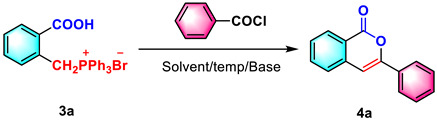					
**Entry**	**Solvent**	**Temp (°C)**	**Base**	**Time**	**Yield (%)**
1	DCM	25	DMAP	12 h	0
2	DCM	25	NEt_3_	12 h	79
3	DCM	25	DBU	12 h	20
4	DCM	25	*t*-BuOK	12 h	10
5	DCM	25	K_2_CO_3_	12 h	50
6	DCM	25	NaOH	12 h	10
7	THF	25	NEt_3_	12 h	38
8	CH_3_CN	25	NEt_3_	12 h	42
9	DCE	25	NEt_3_	12 h	53
10	1,4-dioxane	25	NEt_3_	12 h	57
11	toluene	25	NEt_3_	12 h	77
12	toluene	80	NEt_3_	6 h	80
13	toluene	110	NEt_3_	2 h	82

^a^ (2-carboxybenzyl)triphenylphosphonium bromide **3a** (1.0 mmol), benzoyl chloride (1.1 mmol), base (2.0 mmol), solvent (8.0 mL), 2–12 h; DCM = dichloromethane, DCE = dichloroethane, THF = tetrahydrofuran, DMAP = 4-dimethylaminopyridine, DBU = 1,8-diazabicyclo [5.4.0]undec-7-ene. ^b^ isolated yields.

**Table 2:** Preparation of 1H-isochromen-1-ones **4** ^a^.

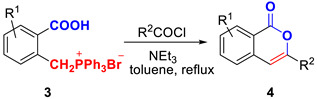									
**Entry**	**R^1^**	**R^2^**	**Time**	**Yield (%)**	**Entry**	**R^1^**	**R^2^**	**Time**	**Yield (%)**
**4a**	H	Ph	2 h	85	**4l**	4-Cl	Ph	2 h	86
**4b**	H	4-CH_3_C_6_H_4_	2 h	84	**4m**	4-Cl	4-CH_3_C_6_H_4_	2 h	84
**4c**	H	4-FC_6_H_4_	2 h	90	**4n**	4-Cl	4-CH_3_OC_6_H_4_	2.5 h	82
**4d**	H	4-CH_3_OC_6_H_4_	2.5 h	83	**4o**	4-Cl	4-*t*-BuC_6_H_4_	2.5 h	81
**4e**	H	3-CH_3_C_6_H_4_	2 h	82	**4p**	4-Cl	3,4,5-(CH_3_O)_3_C_6_H_4_	3 h	80
**4f**	H	4-BrC_6_H_4_	1.5 h	89	**4q**	4-Cl	naphthalen-1-yl	2 h	86
**4g**	H	4-*t*-BuC_6_H_4_	2.5 h	78	**4r**	4-Cl	C_6_H_4_CH=CH	3 h	81
**4h**	H	C_6_H_4_CH=CH	3 h	81	**4s**	4-Cl	2-CH_3_C_6_H_4_	3 h	75
**4i**	H	4-CF_3_C_6_H_4_	1 h	72	**4t**	5-Cl	thiophene-2-yl	2.5 h	80
**4j**	H	2-CH_3_C_6_H_4_	3 h	79	**4u**	5-Cl	CH_3_CH_2_	4 h	60
**4k**	H	naphthalen-1-yl	2 h	85					

^a^ Isolated yields based on the 1H-isochromen-1-ones **4**.

**Table 3:** Linear photophysical parameters of isocoumarin derivatives **4**.

Comps.	Experimental Value					Theoretical Value		
	*λ_a_*	*ε*	*λ_e_*	*∆* *ν*	*Φ*	*λ_calc_*	*f_osc_*	Major Contribution
**4a**	332	1.0	409	5670	1%	338	0.3801	H → L (89%)
	289	3.8				300	0.4871	H → L+1 (85%)
**4h**	365	1.8	427	3978	14%	373	0.9345	H → L (89%)
	322	2.3				326	0.5218	H → L+1 (87%)
	278	3.2				277	0.0204	H-2 → L+1 (61%)
**4j**	335	0.8	404	5098	2%	342	0.4632	H → L (92%)
	290	2.9				305	0.5353	H → L+1 (90%)
**4k**	329	2.6	416	6356	5%	325	0.1213	H → L+1 (92%)
	280	3.2				276	0.0827	H-2 → L+1 (50%)
**4p**	361	1.7	458	5866	12%	367	0.4832	H → L (92%)
	315	2.4				320	0.5485	H → L+1 (87%)
	281	3.1				287	0.3640	H-2 → L+1 (95%)
**4u**	328	0.5	420	6678	---	308	0.1048	H → L (90%)
	276	3.4				266	0.1759	H → L+1 (77%)

*λ_a_*: UV absorption wavelength (nm); *ε*: molar extinction coefficient (×10^4^, L·mol^−1^·cm^−1^); *λ_e_*: emission wavelength (nm); *∆ν*: Stokes shift (cm^−1^); *Φ*: absolute fluorescence quantum yield; *λ_calc_*: theoretical value of absorption wavelength (nm); *f_osc_*: oscillator strength.

## Data Availability

Data are contained within the article and Supplementary Materials.

## Supplementary Materials

The following supporting information can be downloaded at: https://www.mdpi.com/article/10.3390/molecules29112449/s1.

## References

[1] Hussain M., Hussain M.T., Rama N.H., Hameed S., Malik A., Khan K.M. (2003). Synthesis and antimicrobial activities of some isocoumarin and dihydroisocoumarin derivatives. Nat. Prod. Res..

[2] Heynekamp J.J., Hunsaker L.A., Vander Jagt T.A., Royer R.E., Decka L.M., Vander Jagt D.L. (2008). Isocoumarin-based inhibitors of pancreatic cholesterol esterase. Bioorg. Med. Chem..

[3] Nozawa K., Yamada M., Tsuda Y., Kawai K., Nakajima S. (1981). Antifungal activity of oosponol, oospolactone, phyllodulcin, hydrangnol, and some other related compounds. Chem. Pharm. Bull..

[4] Kalinova B., Kindl J., Jiros P., Zacek P., Vasickova S., Budesinsky M., Valterova I. (2009). Composition and electrophysiological activity of constituents identified in male wing gland secretion of the bumblebee parasite aphomia sociella. J. Nat. Prod..

[5] Riveiro M.E., Moglioni A., Vazquez R., Gomez N., Facorro G., Piehl L., De Celis E.R., Shayo C., Davio C. (2008). Structural insights into hydroxycoumarin-induced apoptosis in U-937 cells. Bioorg. Med. Chem..

[6] Shikishima Y., Takaishi Y., Honda G., Ito M., Takeda Y., Kodzhimatov O.K., Ashurmetov O., Lee K.H. (2001). Chemical Constituents of Prangos tschimganica; Structure elucidation and absolute configuration of coumarin and furanocoumarin derivatives with anti-HIV activity. Chem. Pharm. Bull..

[7] Molotkov A.P., Arsenov M.A., Kapustin D.A., Muratov D.V., Shepel N.E., Fedorov Y.V., Smol’yakov A.F., Knyazeva E.I., Lypenko D.A., Dmitriev A.V. (2020). Effect of Cp-ligand methylation on Rhodium(III)-catalyzed annulations of aromatic carboxylic acids with alkynes: Synthesis of isocoumarins and PAHs for organic light-emitting devices. ChemPlusChem.

[8] Arsenov M.A., Fedorov Y.V., Muratov D.V., Nelyubina Y.V., Loginov D.V. (2022). Synthesis of isocoumarins and PAHs with electron-withdrawing substituents: Impact of the substituent nature on the photophysical behavior. Dyes Pigm..

[9] Chutia K., Sarmah M., Gogoi P. (2023). Substituted Isocoumarins: An assemble of synthetic strategies towards 3-substituted and 3,4-disubstituted isocoumarins. Chem. Asian J..

[10] Pirovano V., Marchetti M., Carbonaro J., Brambilla E., Rossi E., Ronda L., Abbiati G. (2020). Synthesis and photophysical properties of isocoumarin-based D-π-A systems. Dyes Pigm..

[11] Han T., Deng H., Yu C.Y.Y., Gui C., Song Z., Kwok R.T.K., Lam J.W.Y., Tang B.Z. (2016). Functional isocoumarin-containing polymers synthesized by rhodium-catalyzed oxidative polycoupling of aryl diacid and internal diyne. Polym. Chem..

[12] Mayakrishnan S., Arun Y., Maheswari N.U., Perumal P.T. (2018). Rhodium(III)-catalysed decarbonylative annulation through C–H activation: Expedient access to aminoisocoumarins by weak coordination. Chem. Commun..

[13] Tian X., Murfin L.C., Wu L.L., Lewis S.E., James T.D. (2021). Fluorescent small organic probes for biosensing. Chem. Sci..

[14] Gogoi N., Parhi R., Tripathi R.K.P., Pachuau L., Kaishap P.P. (2024). Recent advances in synthesis of isocoumarins: An overview. Tetrahedron.

[15] Barry R.D. (1964). Isocoumarins. developments since 1950. Chem. Rev..

[16] Oliver M.A., Gandour R.D. (1984). The identity of 4-bromo-3-phenylisocoumarin. A facile preparation by bromolactonization of alkyl 2-(2-phenylethynyl)benzoates. J. Org. Chem..

[17] Pal S., Chatare V., Pal M. (2011). Isocoumarin and its derivatives: An overview on their synthesis and applications. Curr. Org. Chem..

[18] Zhang M.L., Zhang H.J., Han T.T., Ruan W.Q., Wen T.B. (2015). Rh(III)-catalyzed oxidative coupling of benzoic acids with geminal substituted vinyl acetates: Synthesis of 3-substituted isocoumarins. J. Org. Chem..

[19] Cai S.J., Wang F., Xi C.J. (2012). Assembly of 3-substituted isocoumarins via a CuI-catalyzed domino coupling/addition/deacylation process. J. Org. Chem..

[20] Gao Q.C., Li Y.F., Xuan J., Hu X.Q. (2023). Practical synthesis of isocoumarins via Rh(III)-catalyzed C–H activation/annulation cascade. Beilstein J. Org. Chem..

[21] Zhang Q., Zhang L.Y., Shi X.Y. (2023). Copper-promoted intramolecular oxidative dehydrogenation for synthesizing dihydroisocoumarins and isocoumarins. Molecules.

[22] Wang Y.H., Qiu G., Zhou H., Xie W., Liu J.B. (2019). Regioselective cyclization of 2-alkynylbenzoic acid in water for the synthesis of isocoumarin. Tetrahedron.

[23] Jang Y.J., Chen G.Y., Jhan Y.L., Lo P.T., Hsu W.Y., Wang K., Hsu Y.T., Lee C.L., Yang Y.L., Wu Y.C. (2023). Chemo- and regioselective construction of functionalized isocoumarin, flavone, and isoquinolinedione via a one-pot reaction of o-quinol acetate and soft nucleophiles. Adv. Synth. Catal..

[24] Chen Z., Nieves-Quinones Y., Waas J.R., Singleton D.A. (2014). Isotope effects, dynamic matching, and solvent dynamics in a Wittig reaction. betaines as bypassed intermediates. J. Am. Chem. Soc..

[25] Zeng X.P., Cao Z.Y., Wang X., Chen L., Zhou F., Zhu F., Wang C.H., Zhou J. (2016). Activation of chiral (salen)AlCl complex by phosphorane for highly enantioselective cyanosilylation of ketones and enones. J. Am. Chem. Soc..

[26] Kim J.K., Liu Y.B., Gong M., Li Y.B., Huang M.M., Wu Y.J. (2023). A facile visible-light-induced one-pot synthesis of 3-alkyl coumarins from simple salicylaldehydes. Tetrahedron.

[27] Lee C.J., Chang T.H., Yu J.K., Reddy G.M., Hsiao M.Y., Lin W.W. (2016). Synthesis of functionalized furans via chemoselective reduction/Wittig reaction using catalytic triethylamine and phosphine. Org. Lett..

[28] Saleh N., Voituriez A. (2016). Synthesis of 9H-pyrrolo[1,2-a]indole and 3H-pyrrolizine derivatives via a phosphine-catalyzed umpolung addition/intramolecular Wittig reaction. J. Org. Chem..

[29] Longwitz L., Spannenberg A., Werner T. (2019). Phosphetane oxides as redox cycling catalysts in the catalytic Wittig reaction at room temperature. ACS Catal..

[30] Schneider L.M., Schmiedel V.M., Pecchioli T., Lentz D., Merten C., Christmann M. (2017). Asymmetric synthesis of carbocyclic propellanes. Org. Lett..

[31] Grandane A., Longwitz L., Roolf C., Spannenberg A., Escobar H.M., Junghanss C., Suna E., Werner T. (2018). Intramolecular base-free catalytic Wittig reaction: Synthesis of benzoxepinones. J. Org. Chem..

[32] Chien P.C., Chen Y.R., Chen Y.J., Chang C.F., Marri G., Lin W.W. (2023). Synthesis of Furo[2,3-f]dibenzotropones via Intramolecular Wittig Reaction of Alkylidene Dibenzo-β-tropolones. Adv. Synth. Catal..

[33] Sun M., Wan Q., Ding M.W. (2019). New facile synthesis of furan-2(3H)-ones and 2,3,5-trisubstituted furans via intramolecular Wittig reaction of acid anhydride. Tetrahedron.

[34] Kayser M.M., Bxeau L. (1988). Neighboring effects on regioselectivity of Wittig reactions with maleic anhydrides. Tetrahedron Lett..

[35] Wang L., Ren Z.L., Ding M.W. (2015). Synthesis of 2,3-dihydro-1H-2-benzazepin-1-ones and 3H-2-benzoxepin-1-ones by isocyanide-based multicomponent reaction/Wittig sequence starting from phosphonium salt precursors. J. Org. Chem..

[36] Zeng X.H., Wang H.M., Ding M.W. (2015). Unexpected synthesis of 5,6-dihydropyridin-2(1H)-ones by a domino Ugi/aldol/hydrolysis reaction starting from baylis–hillman phosphonium Salts. Org. Lett..

[37] Ren Z.L., Guan Z.R., Kong H.H., Ding M.W. (2017). Multifunctional odorless isocyano(triphenylphosphoranylidene)-acetates: Synthesis and direct one-pot four-component Ugi/Wittig cyclization to multisubstituted oxazoles. Org. Chem. Front..

[38] Yan Y.M., Rao Y., Ding M.W. (2017). One-pot synthesis of indoles by a sequential Ugi-3CR/Wittig reaction starting from odorless isocyanide-substituted phosphonium salts. J. Org. Chem..

[39] Sun M., Zhao L., Ding M.W. (2019). One-pot-three-component synthesis of 2-(1,2,3,4-tetrahydroisoquinolin-1-yl)oxazoles via DEAD-promoted oxidative Ugi/Wittig reaction. J. Org. Chem..

[40] Xiong J., Zhi Y.M., Yao G., Zhang J.A., Feng Q.X., He H.T., Pang Y.L., Shi H., Ding M.W. (2021). One-pot synthesis of polysubstituted pyrroles via sequential ketenimine formation/Ag(I)-catalyzed alkyne cycloisomerisation starting from ylide adducts. Chin. J. Chem..

[41] Zeng C.Y., Cao Z., He Y.R., Ye T.T., Gao Y.S., Li D.H., Liu Q.M., Zhou W.W., Fang W.Y. (2022). Multi-stimuli-responsive fluorescence of bibranched bromo-substituted cyanostilbene derivative with aggregation induced emission enhancement and green light-emitting diode. Results Opt..

[42] Zeng C.Y., Dai J., Yang T.S., Wang Z.J., Gao Y., Xia J., Chen Y., Sun M. (2024). Multi-stimuli fluorescence responsiveness of α-cyanostilbene derivative: AIEE, stimuli response to polarity, acid, force and light, applications in anti-counterfeiting and single phosphor w-OLED. Dyes Pigm..

[43] Ren T.B., Xu W., Zhang W., Zhang X.X., Wang Z.Y., Zhen X., Lin Y., Zhang X.B. (2018). A general method to increase stokes shift by introducing alternating vibronic structures. J. Am. Chem. Soc..

[44] Fang W.L., Liang Z.Y., Guo X.F., Wang H. (2024). A D-π-A-based near-infrared fluorescent probe with large Stokes shift for the detection of cysteine in vivo. Talanta.

[45] Wang C.L., Dong H.L., Jiang L., Hu W.P. (2018). Organic semiconductor crystals. Chem. Soc. Rev..

[46] Jiang G.B., Xiao Li J.X., Zhu C.L., Wu W.Q., Jiang H.F. (2017). Palladium-Catalyzed Sequential Nucleophilic Addition/Oxidative Annulation of Bromoalkynes with Benzoic Acids to Construct Functionalized Isocoumarins. Org. Lett..

[47] Kumar M.R., Irudayanathan F.M., Moon J.H., Lee S. (2013). Regioselective One-Pot Synthesis of Isocoumarins and Phthalides from 2-Iodobenzoic Acids and Alkynes by Temperature Control. Adv. Synth. Catal..

