# Supplementary material for: Novel Metal-Free Synthesis of 3-Substituted Isocoumarins and Evaluation of Their Fluorescence Properties for Potential Applications
Source: Molecules. 2024 May 23;29(11):2449. doi: 10.3390/molecules29112449 (PMC11173990; doi:10.3390/molecules29112449)

Supporting Information

Novel Metal-Free Synthesis of 3-Substituted Isocoumarins and Evaluation of Their Fluorescence Properties for Potential Applications

Mei Sun,[1,2†] Chong-Yang Zeng,[1†] Lu-Lu Bu,[1] Mai Xu,[1] Kai Chen,[1] Jia-Lin Liu,[1] Tao Zhang,[1] Jia-You Dai,[1] Jia-Xin Hong,[*,3] Ming-Wu Ding[*,2]

[1] School of Chemistry and Materials Engineering, Huainan Normal University, Huainan 232038, Anhui, China.

[2] Key Laboratory of Pesticide & Chemical Biology of Ministry of Education, Hubei International Scientific and Technological Cooperation Base of Pesticide and Green Synthesis, Central China Normal University, Wuhan 430079, P. R. China.

[3] College of Food Science and Engineering, Jiangxi Agricultural University, Nanchang 330045, China

*Corresponding author. E-mail: lchongjiaxin@163.com (Jia-Xin Hong); mwding@mail.ccnu.edu.cn (Ming-Wu Ding).

Table of Contents

1. Copies of ^{1}H and ^{13}C NMR spectrum of compound **4a-4u** ……………………..……..……………2-22

^{1}H NMR (600 MHz, $CDCl_3$) of compound **4a**

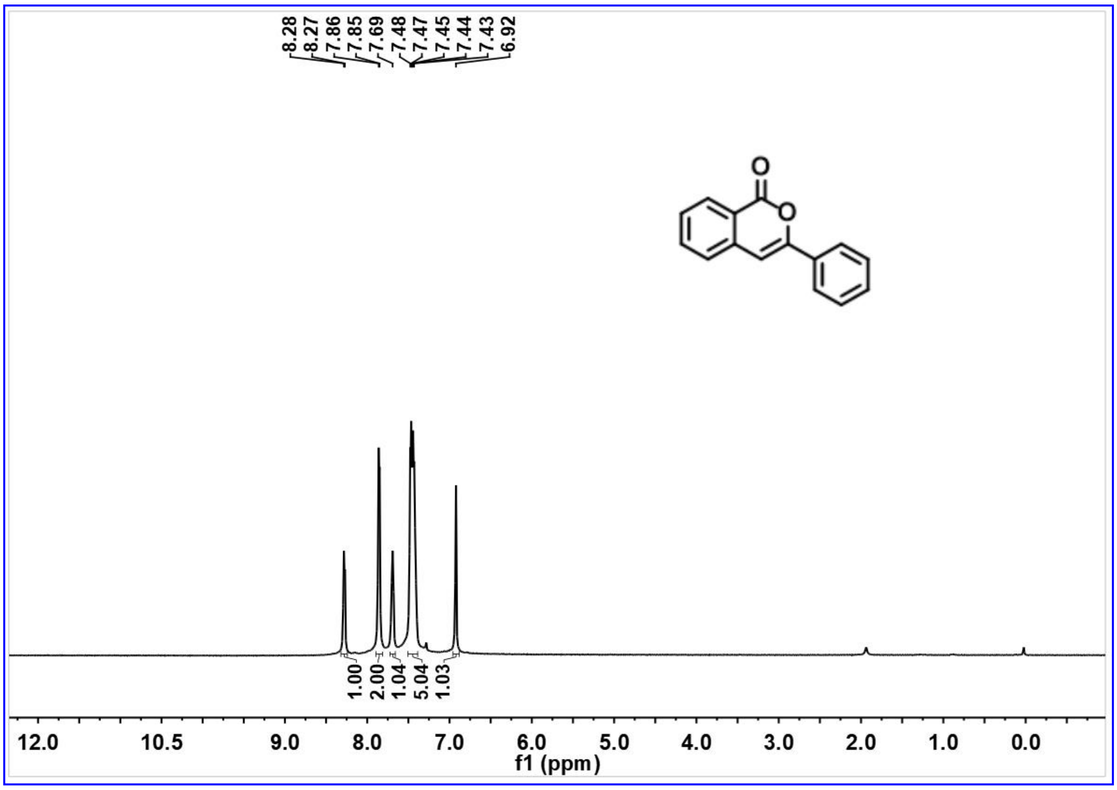

$^{13}C\{^{1}H\}$ NMR (150 MHz, $CDCl_3$) of compound **4a**

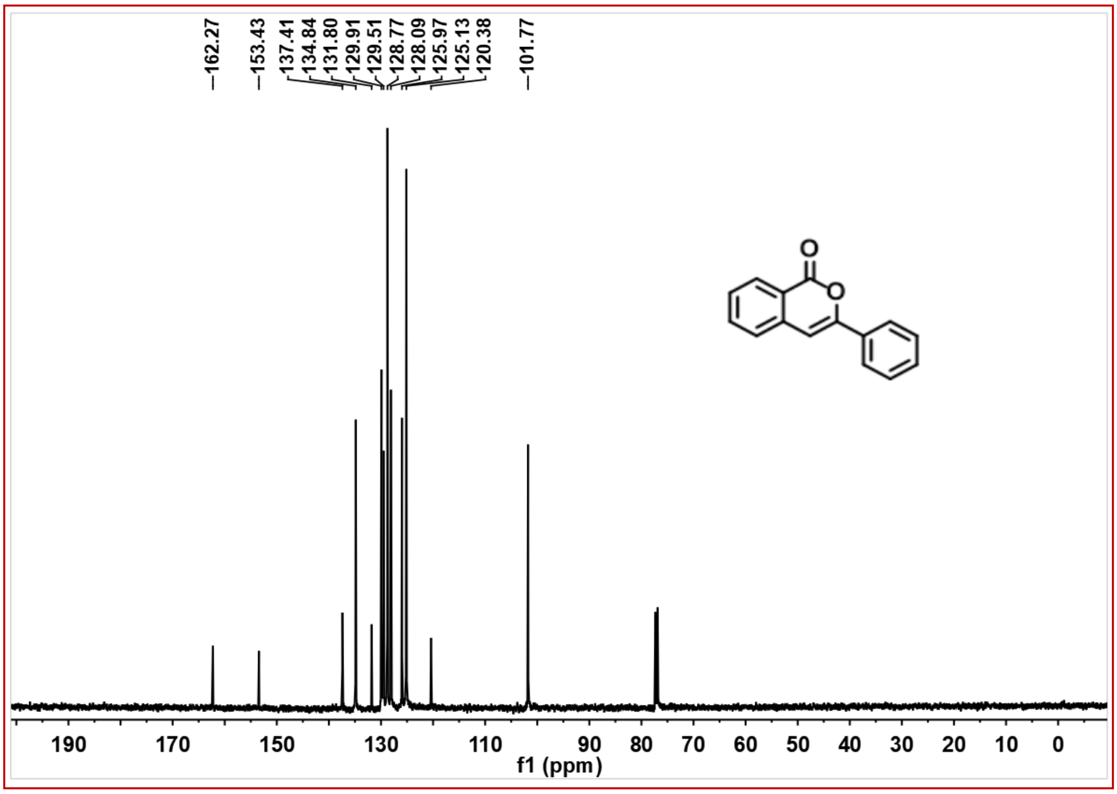

^{1}H NMR (600 MHz, $CDCl_3$) of compound **4b**

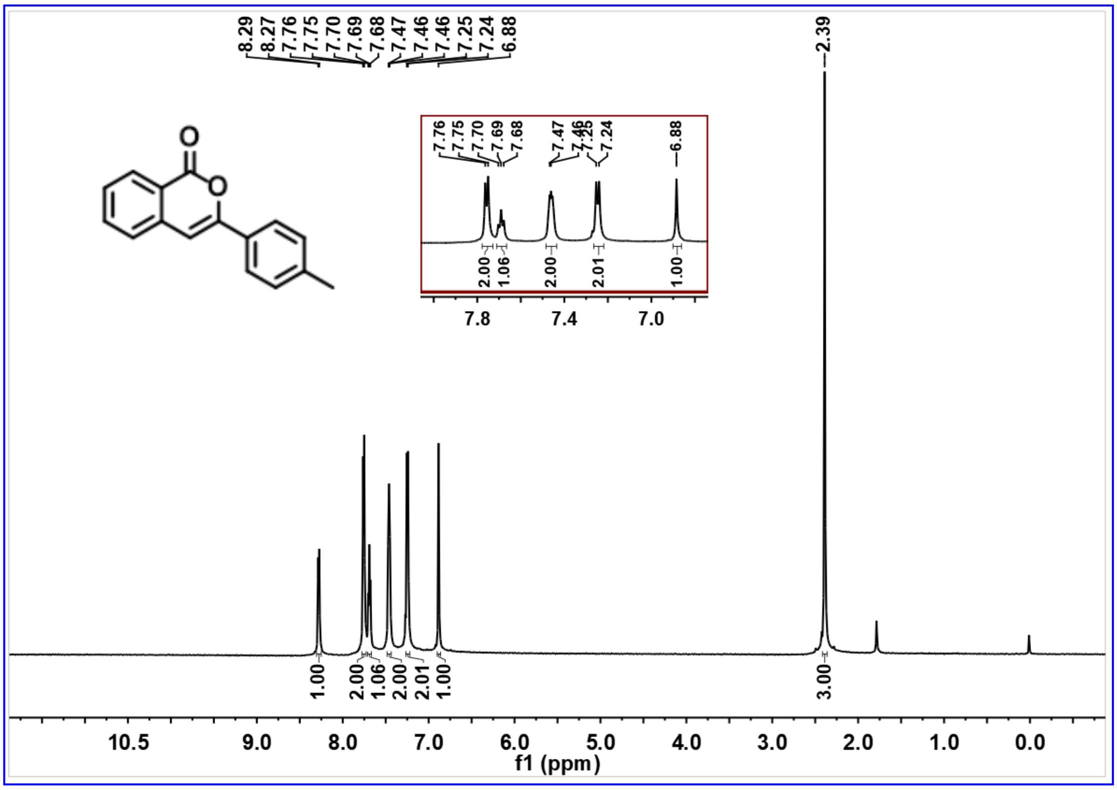

$^{13}C\{^{1}H\}$ NMR (150 MHz, $CDCl_3$) of compound **4b**

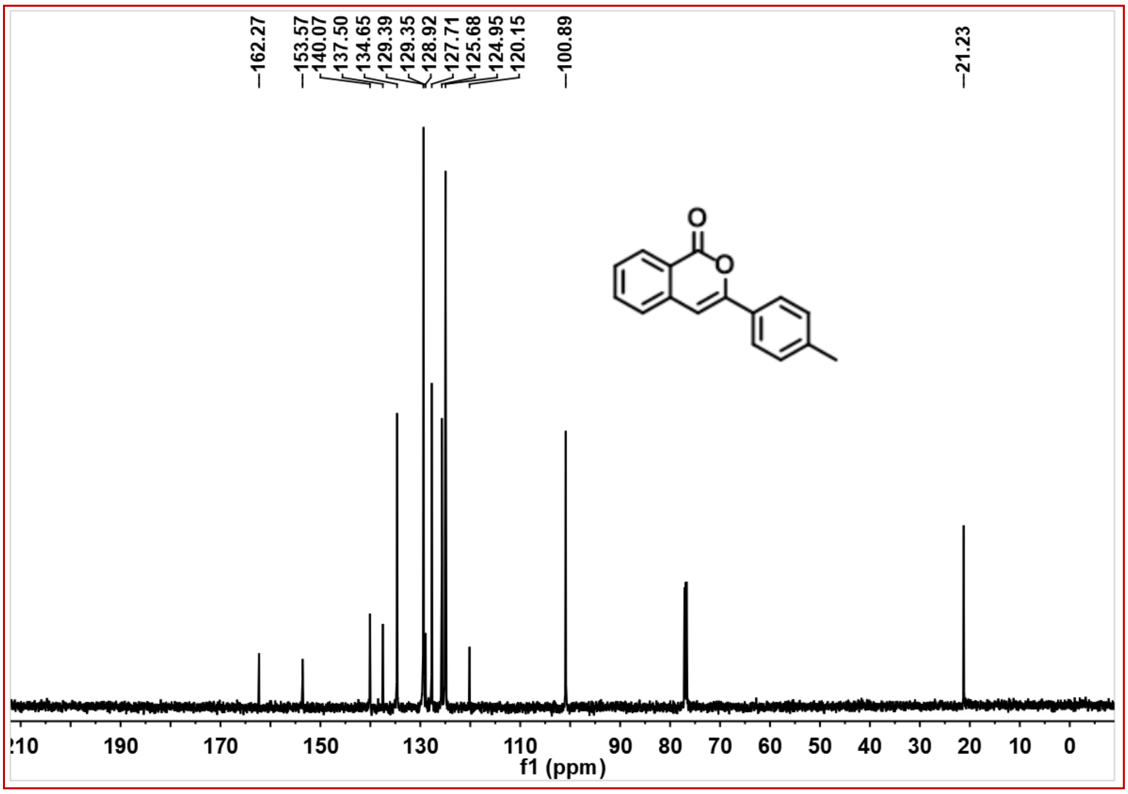

^{1}H NMR (600 MHz, $CDCl_3$) of compound **4c**

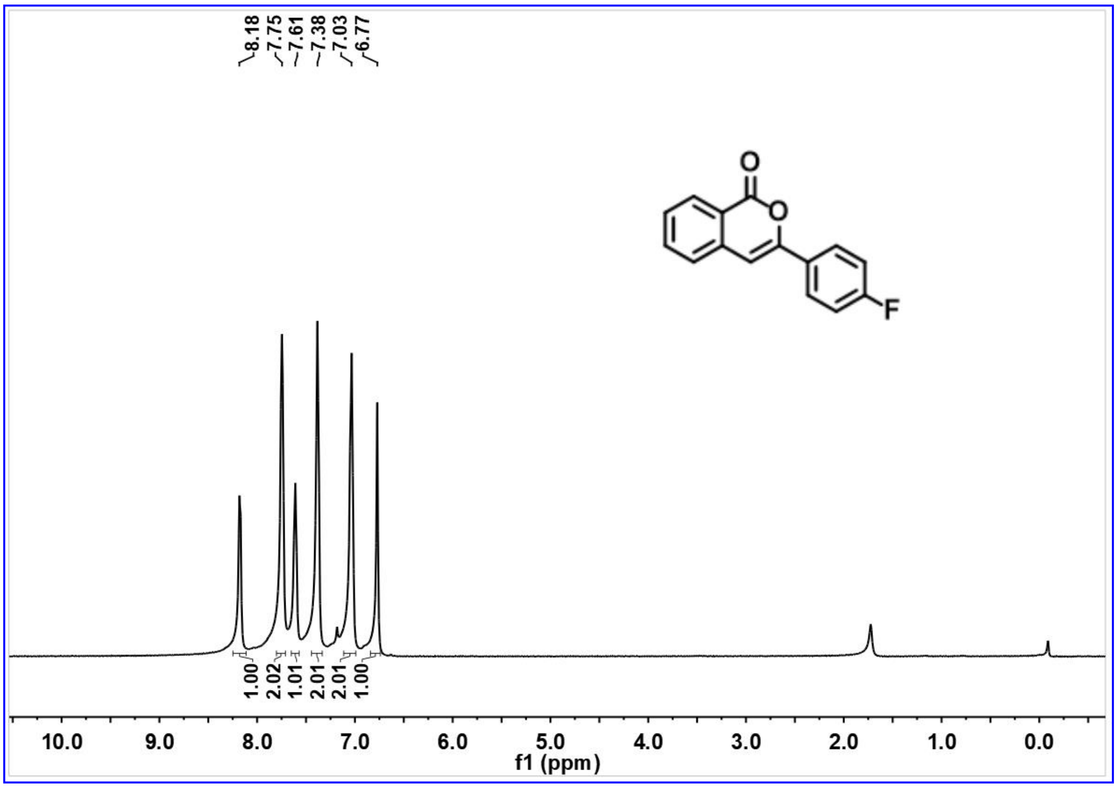

$^{13}C\{^{1}H\}$ NMR (150 MHz, $CDCl_3$) of compound **4c**

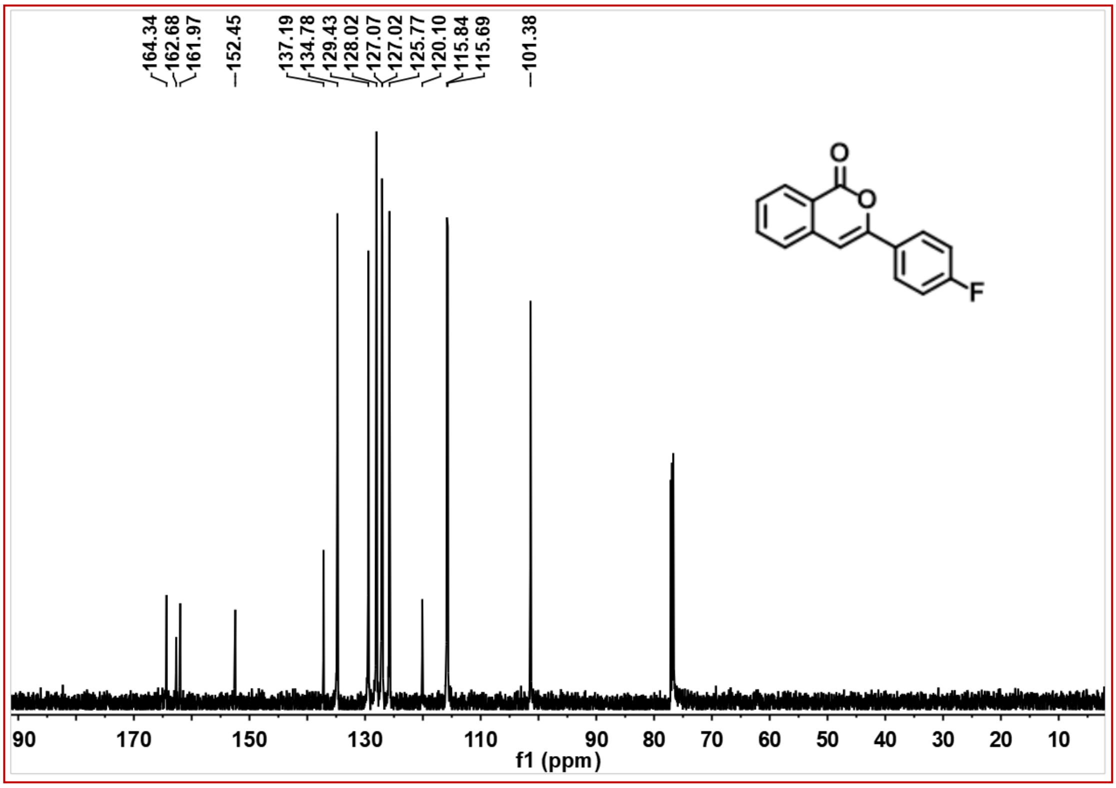

^{1}H NMR (600 MHz, $CDCl_3$) of compound **4d**

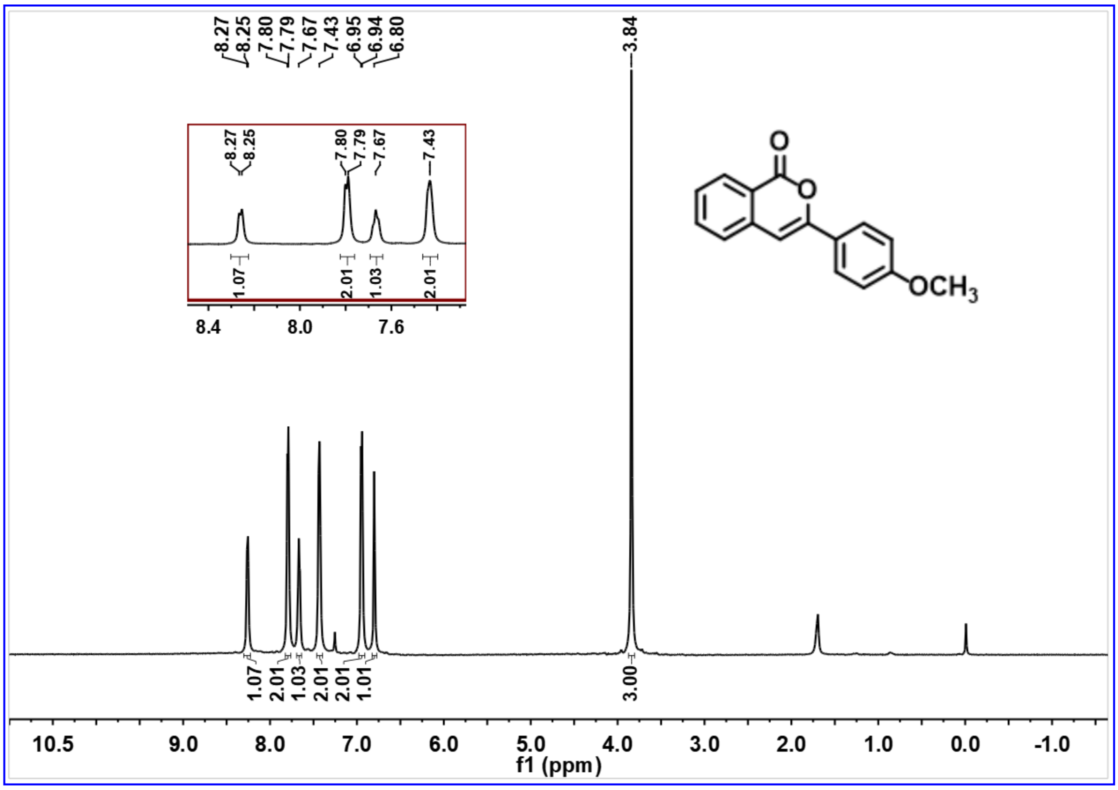

^{13}C{^{1}H} NMR (150 MHz, $CDCl_3$) of compound **4d**

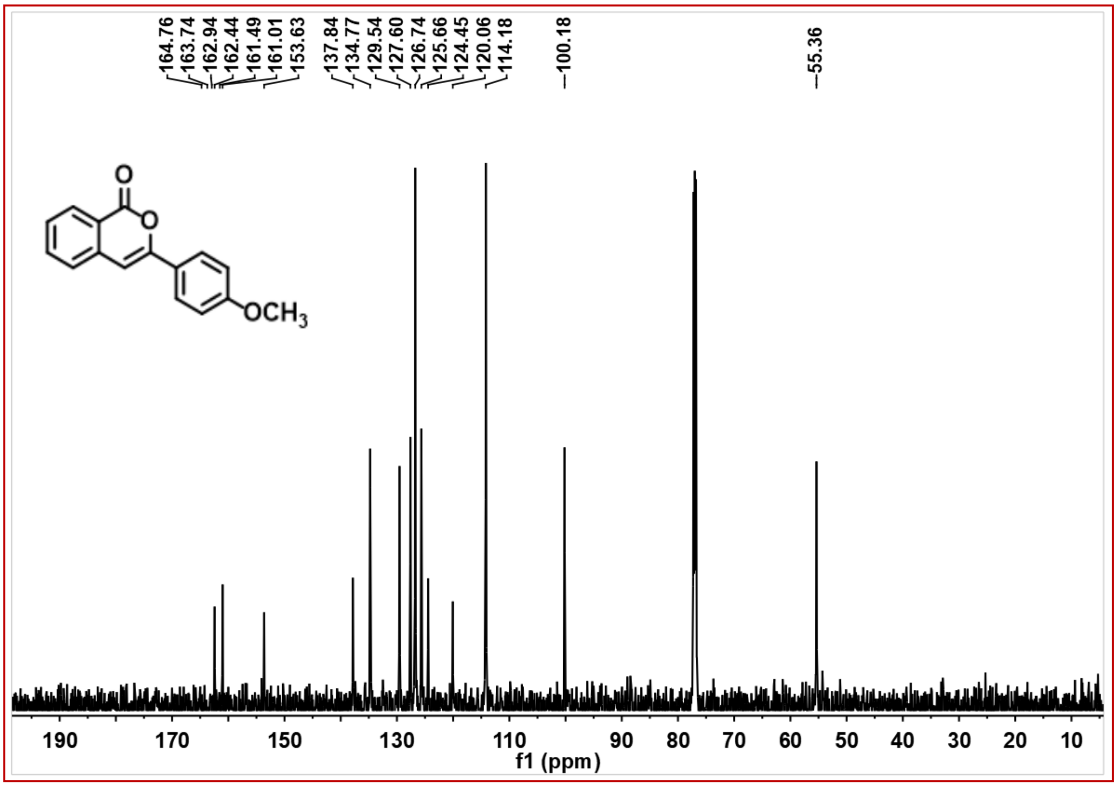

^{1}H NMR (600 MHz, $CDCl_3$) of compound **4e**

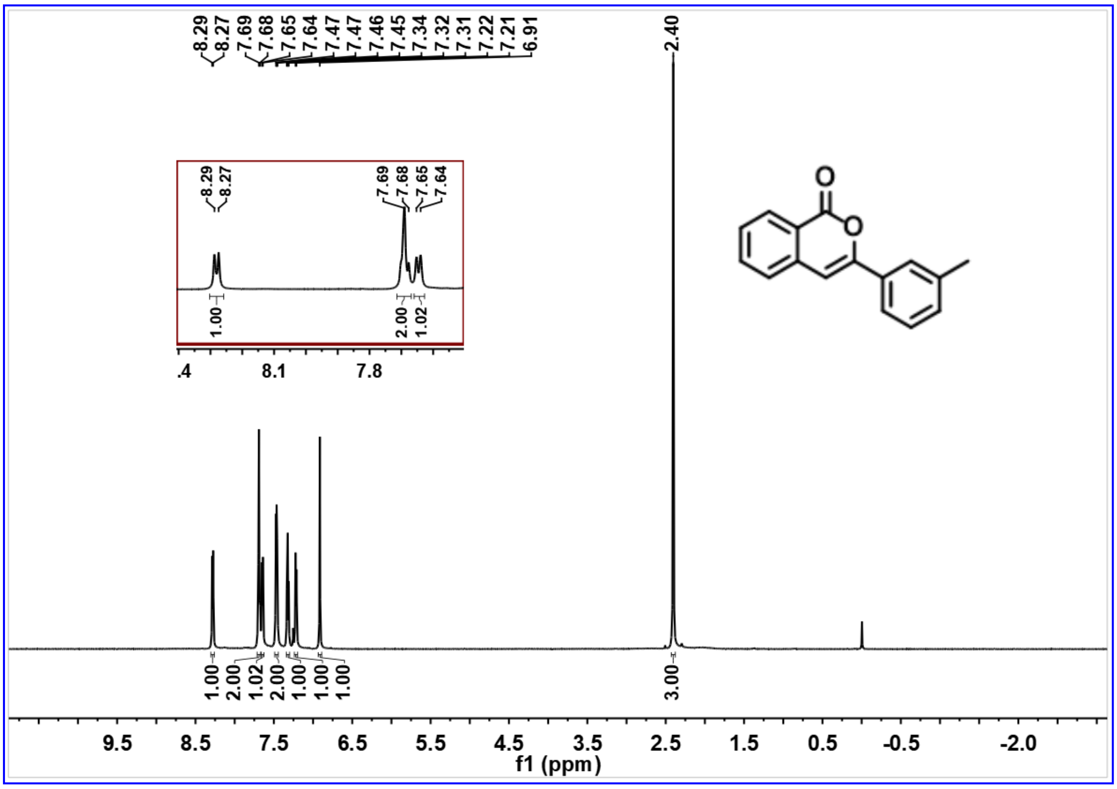

^{13}C{^{1}H} NMR (150 MHz, $CDCl_3$) of compound **4e**

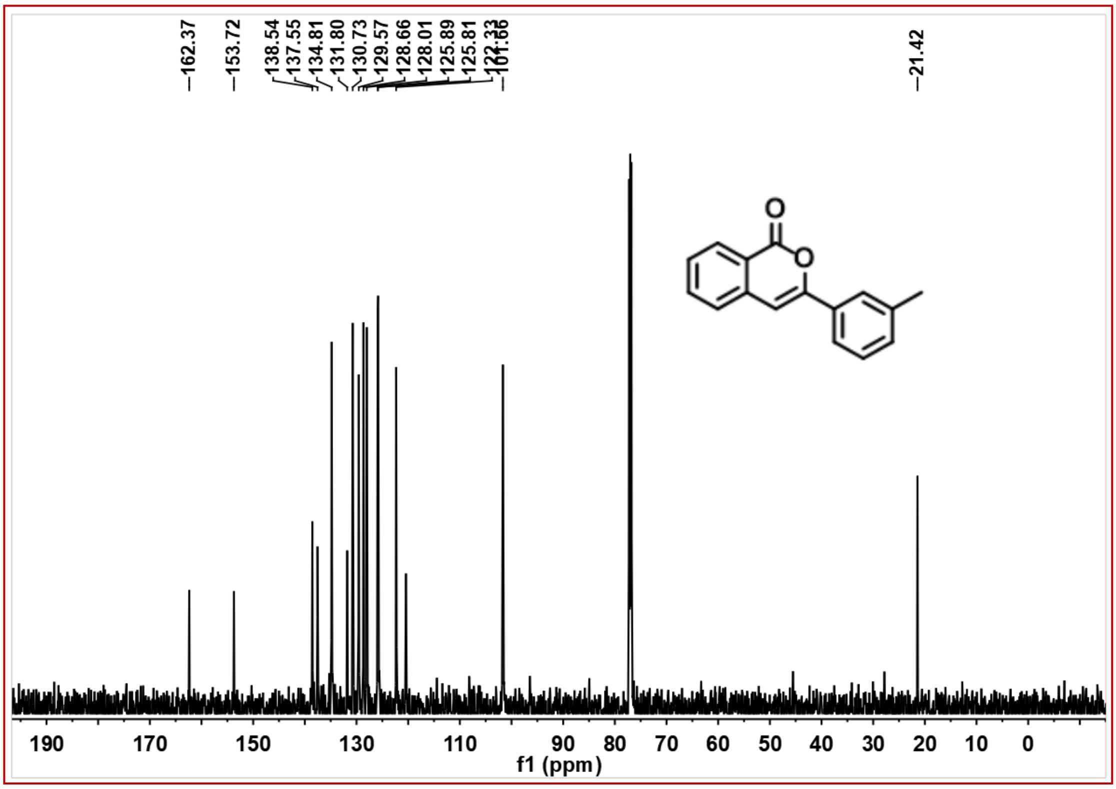

^{1}H NMR (600 MHz, $CDCl_3$) of compound **4f**

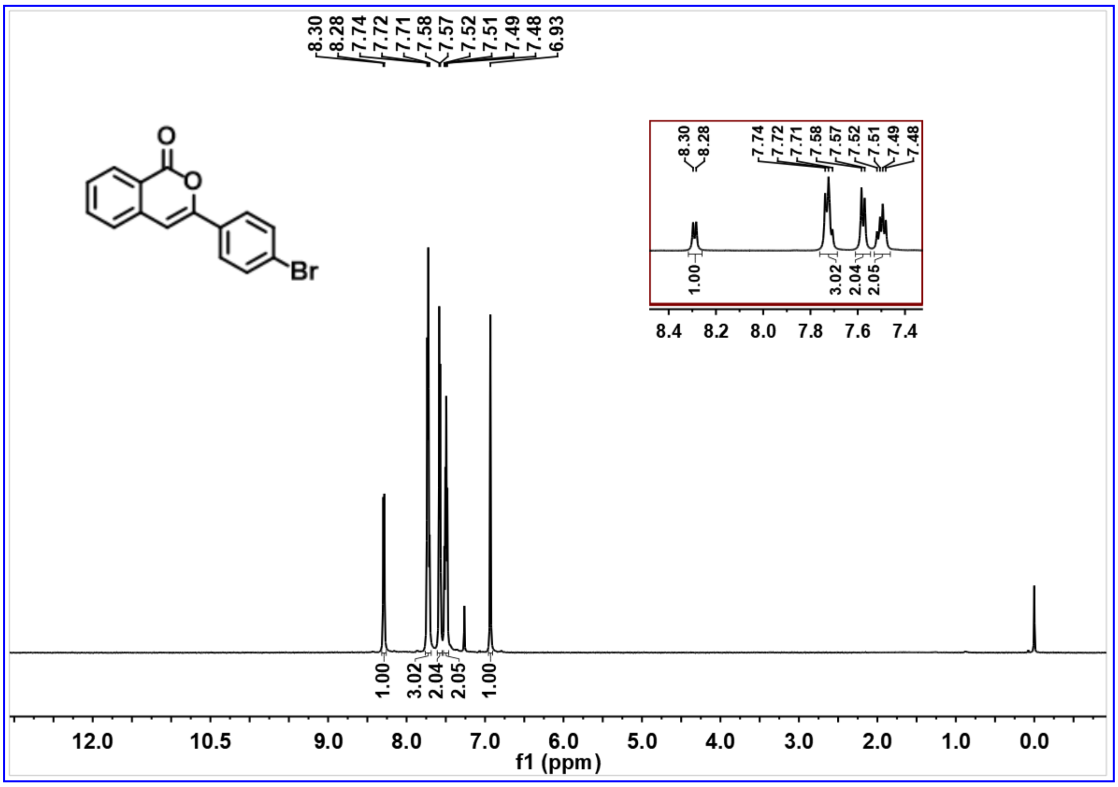

$^{13}C\{^{1}H\}$ NMR (150 MHz, $CDCl_3$) of compound **4f**

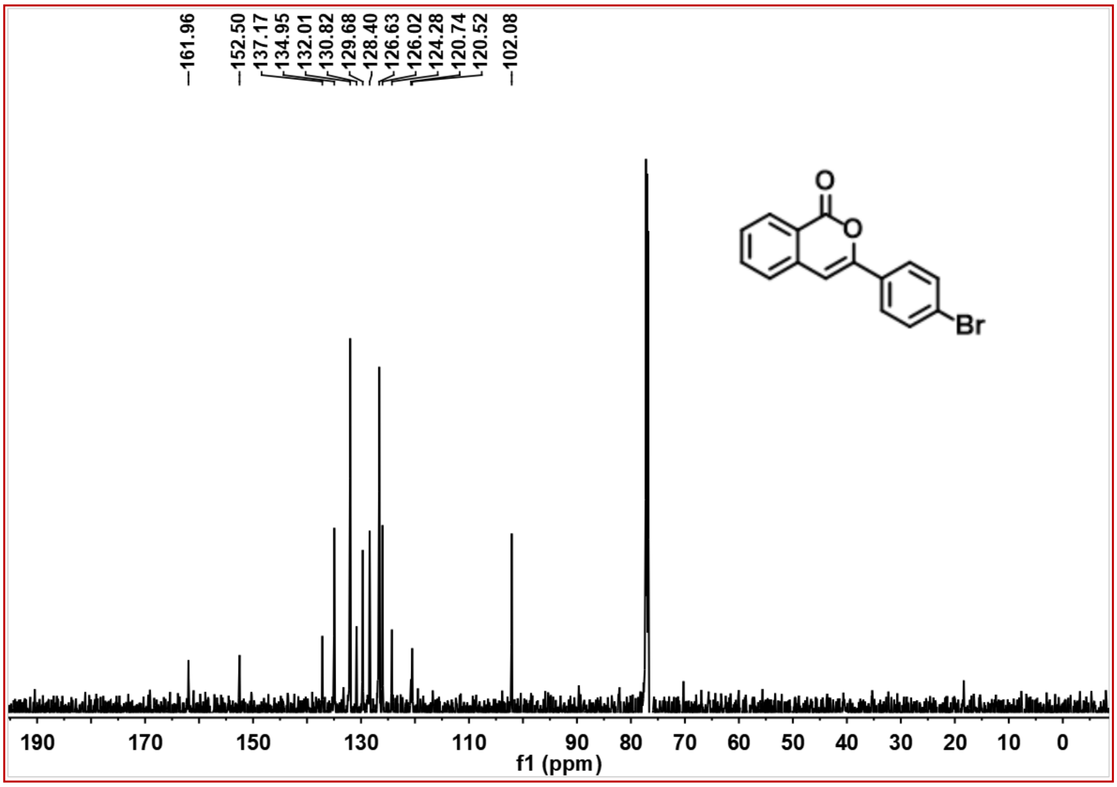

^{1}H NMR (600 MHz, $CDCl_3$) of compound **4g**

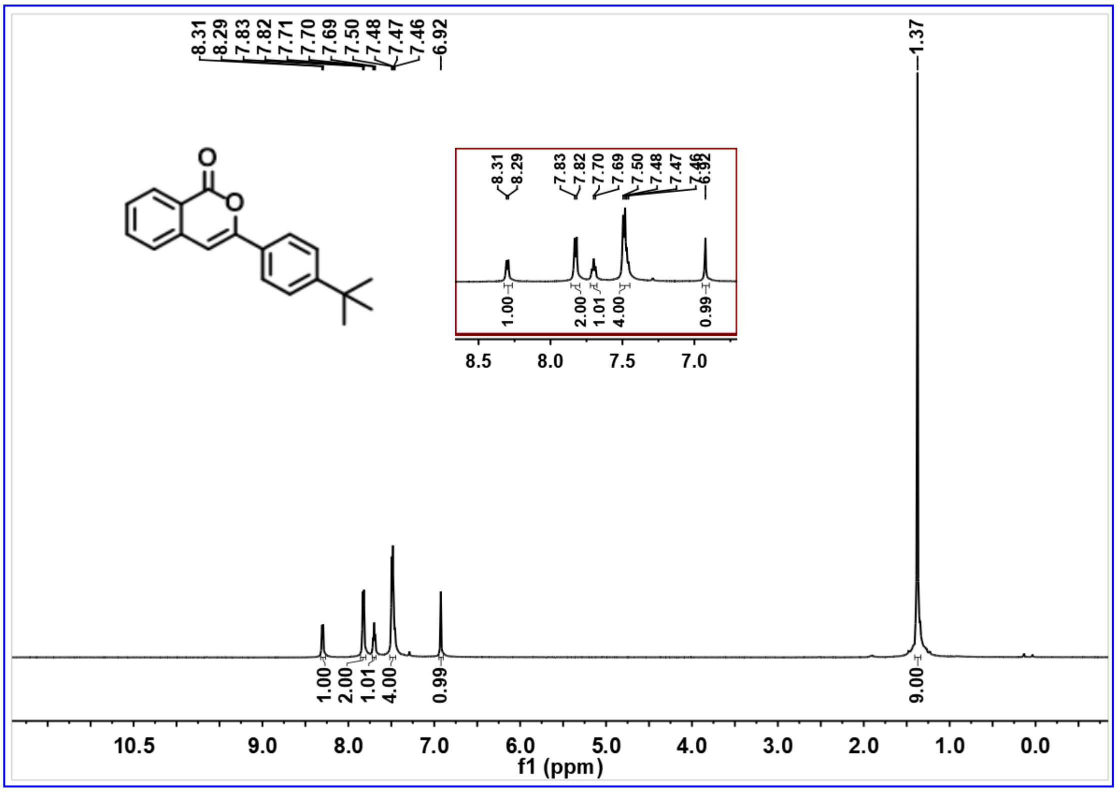

$^{13}C\{^{1}H\}$ NMR (150 MHz, $CDCl_3$) of compound **4g**

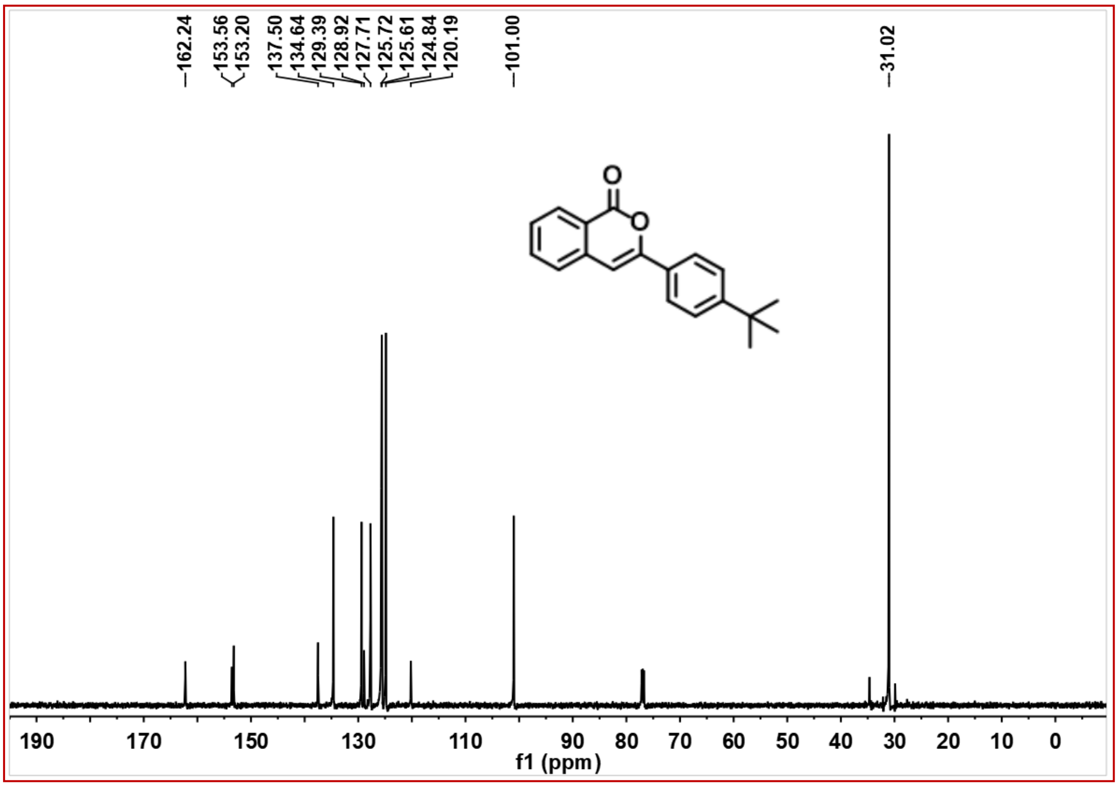

^{1}H NMR (600 MHz, $CDCl_3$) of compound **4h**

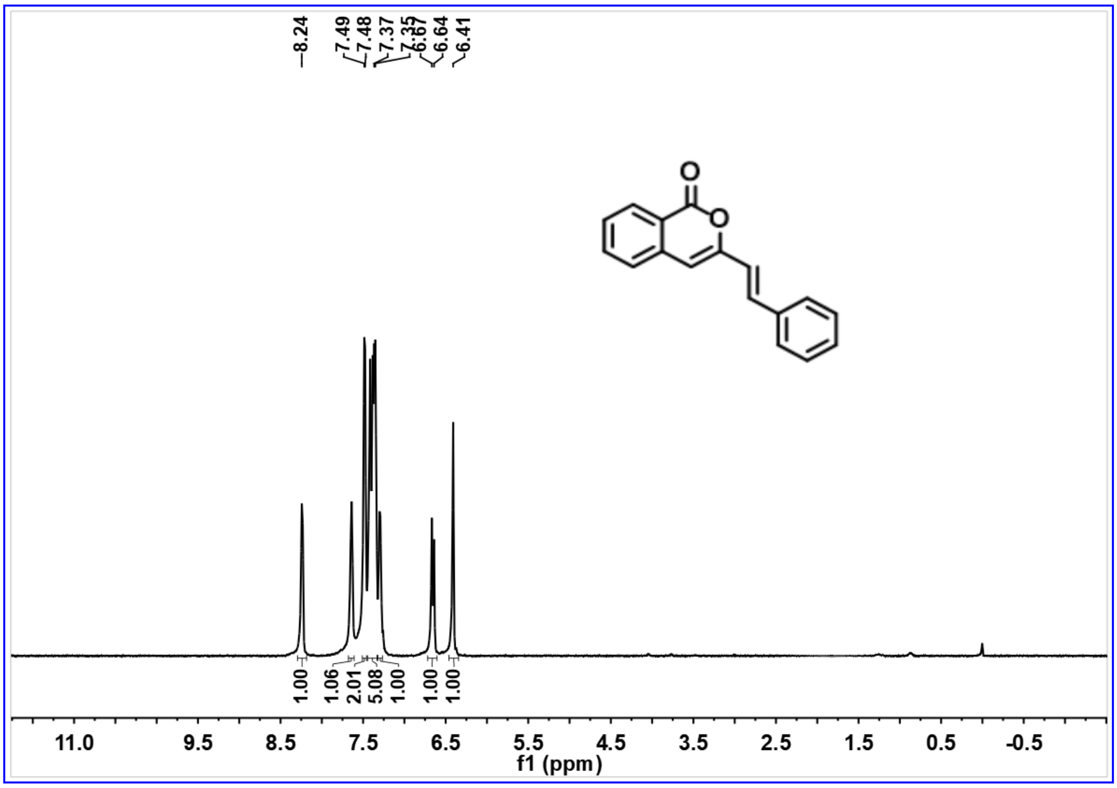

$^{13}C\{^{1}H\}$ NMR (150 MHz, $CDCl_3$) of compound **4h**

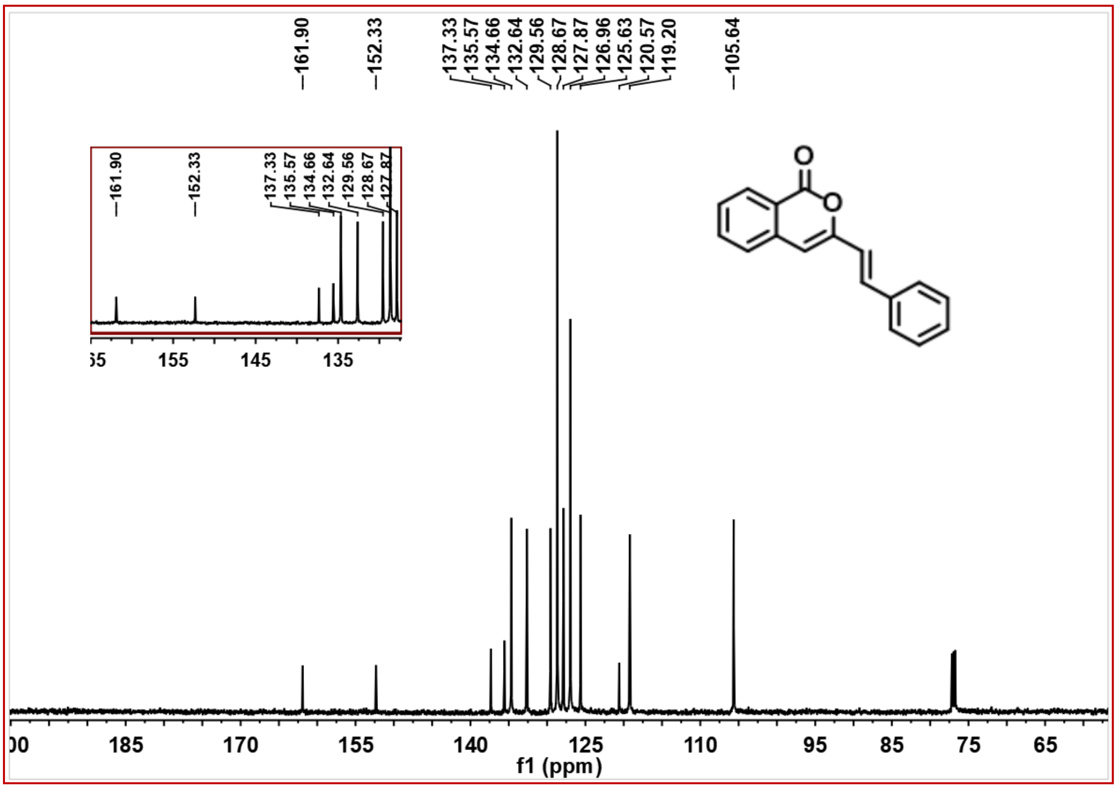

^{1}H NMR (600 MHz, $CDCl_3$) of compound **4i**

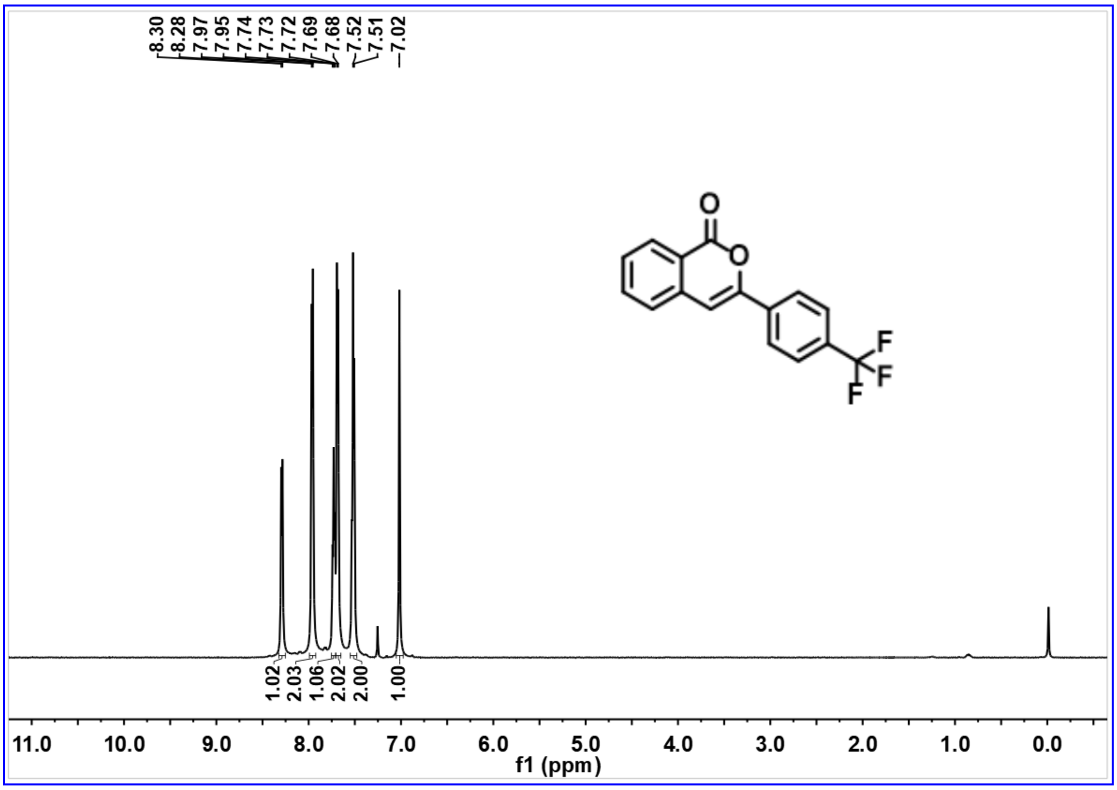

$^{13}C\{^{1}H\}$ NMR (150 MHz, $CDCl_3$) of compound **4i**

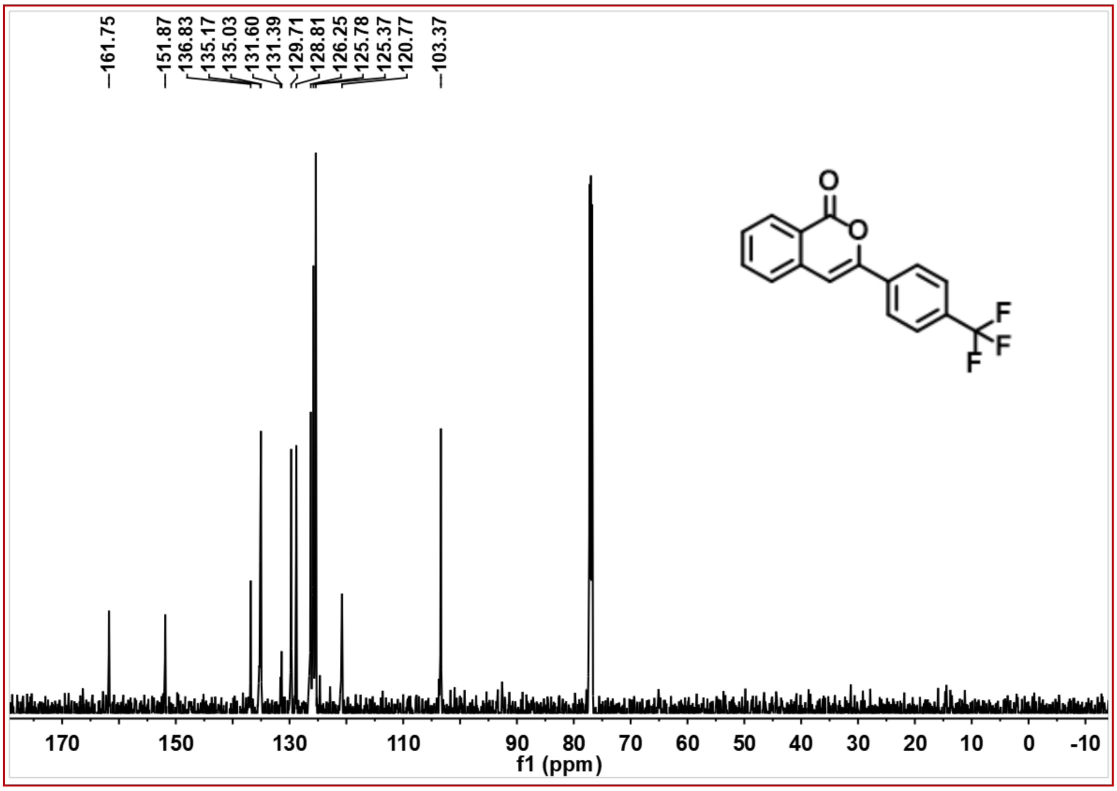

^{1}H NMR (600 MHz, $CDCl_3$) of compound **4j**

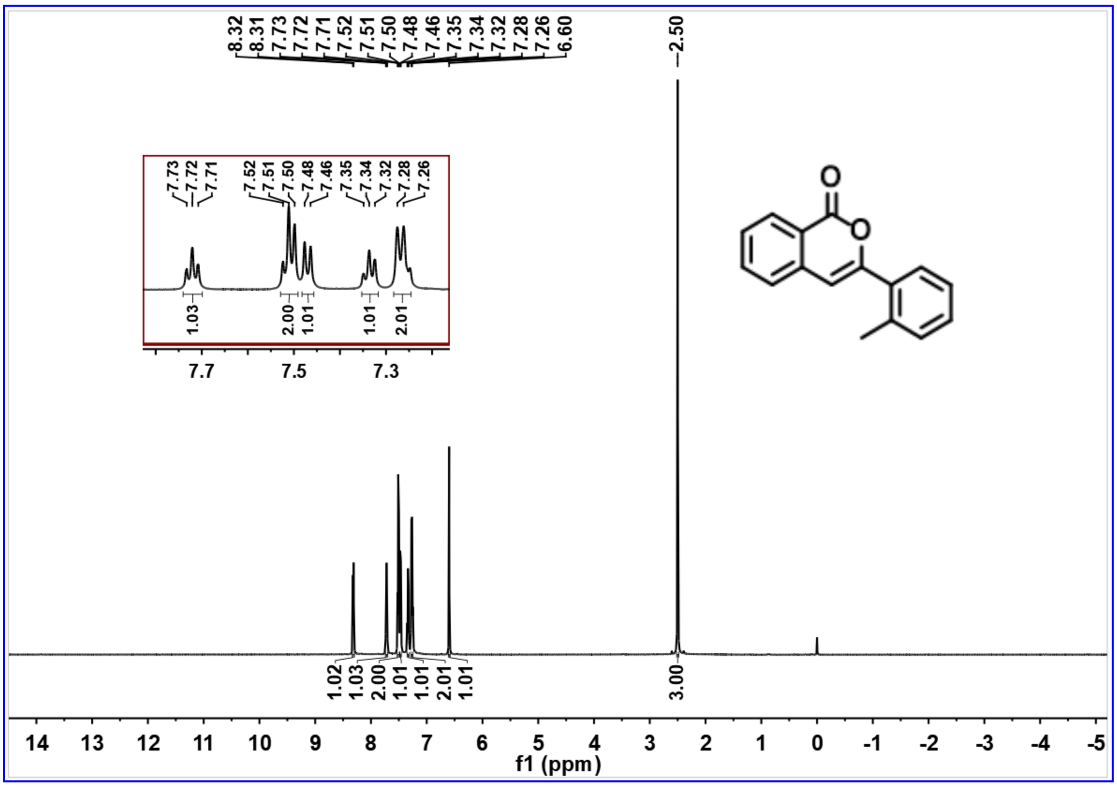

$^{13}C\{^{1}H\}$ NMR (150 MHz, $CDCl_3$) of compound **4j**

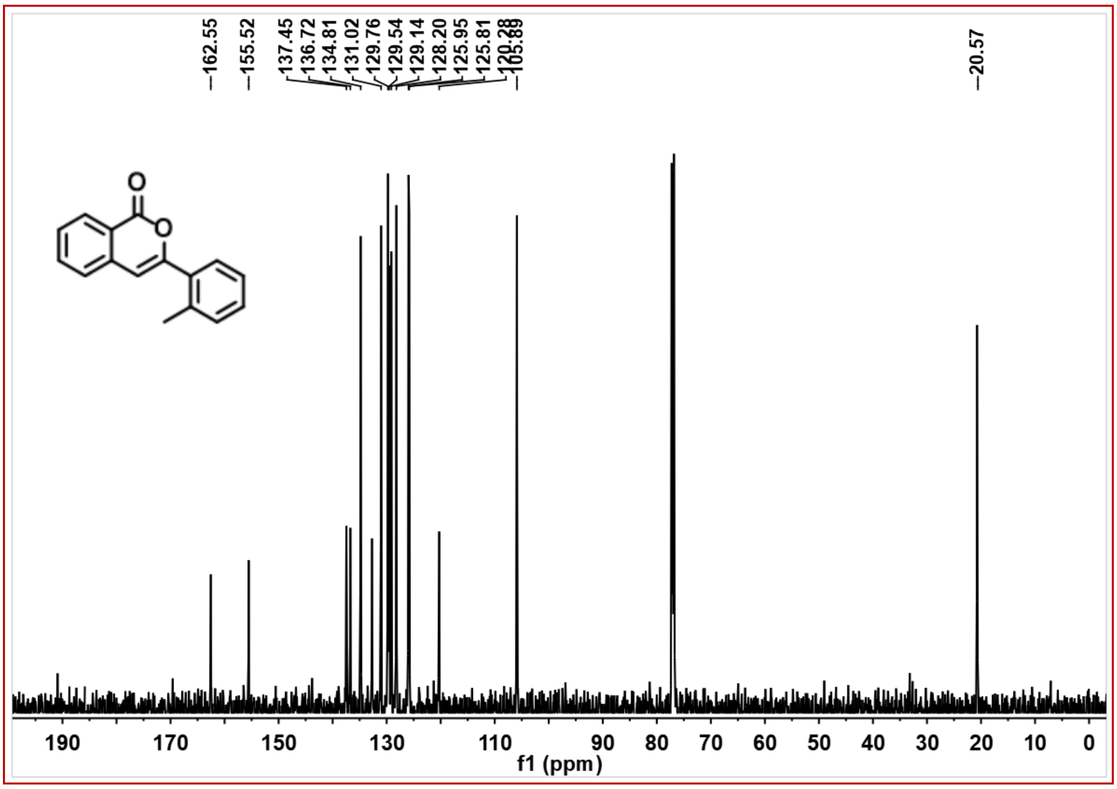

^{1}H NMR (600 MHz, $CDCl_3$) of compound **4k**

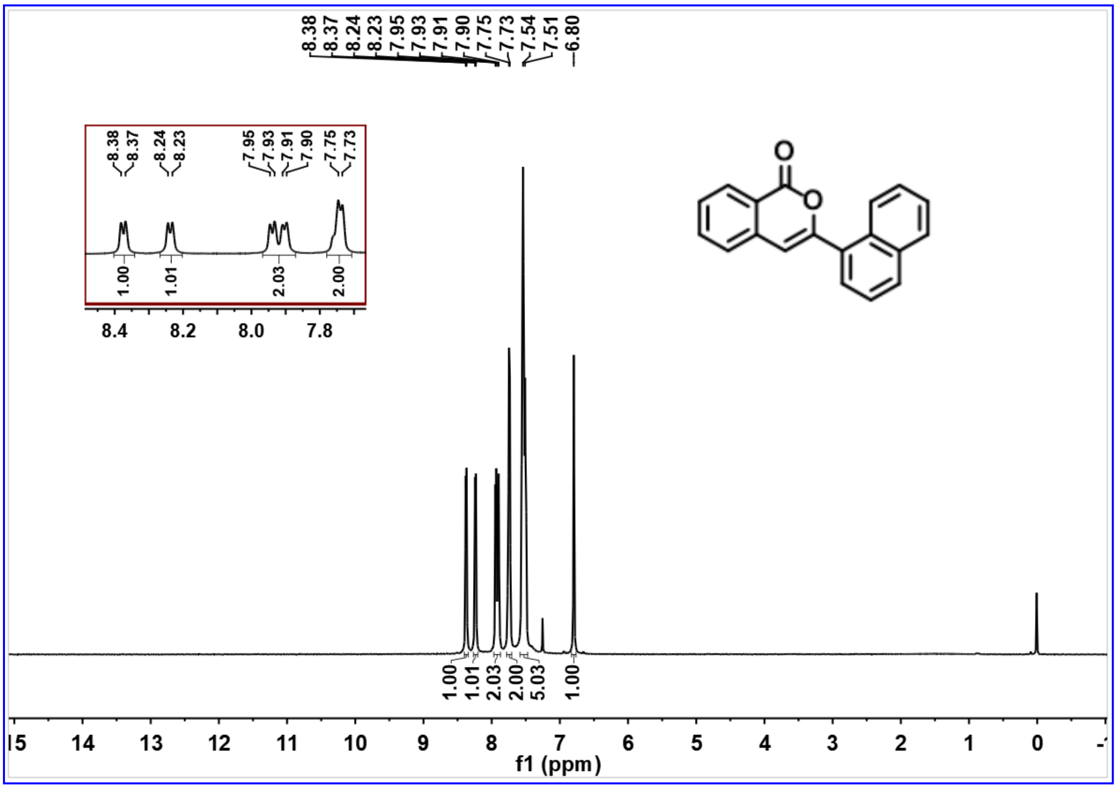

$^{13}C\{^{1}H\}$ NMR (150 MHz, $CDCl_3$) of compound **4k**

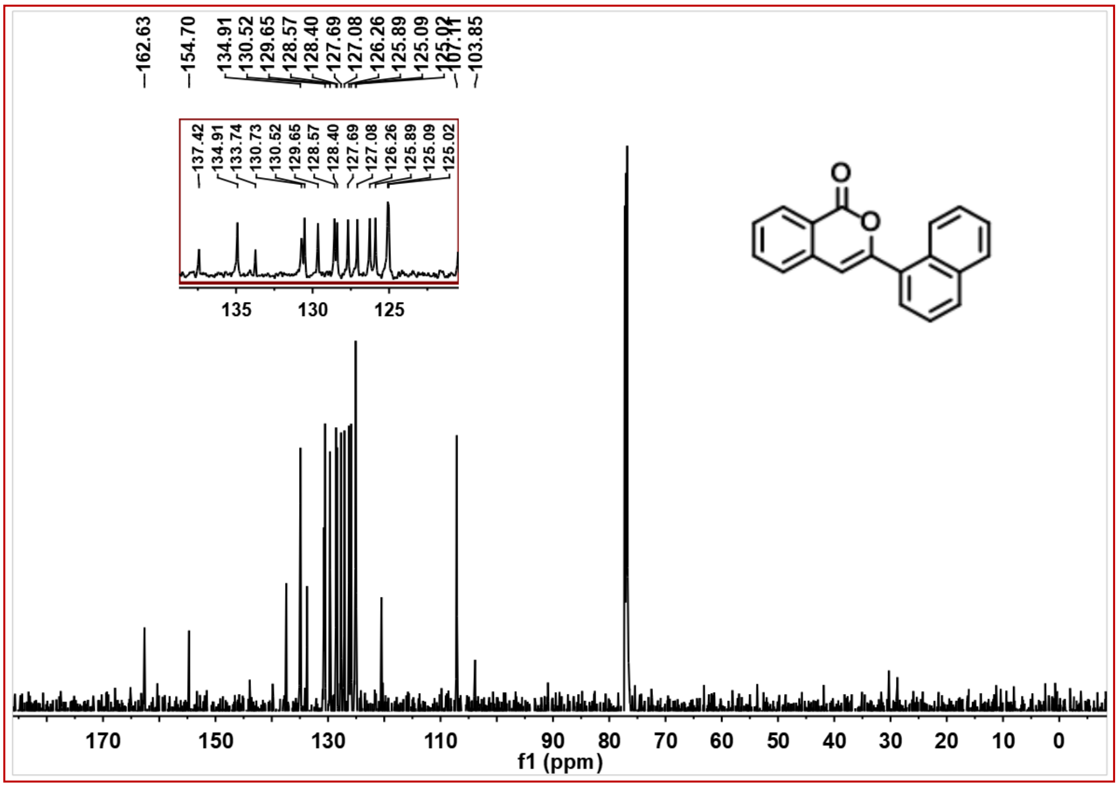

^{1}H NMR (600 MHz, $CDCl_3$) of compound **4l**

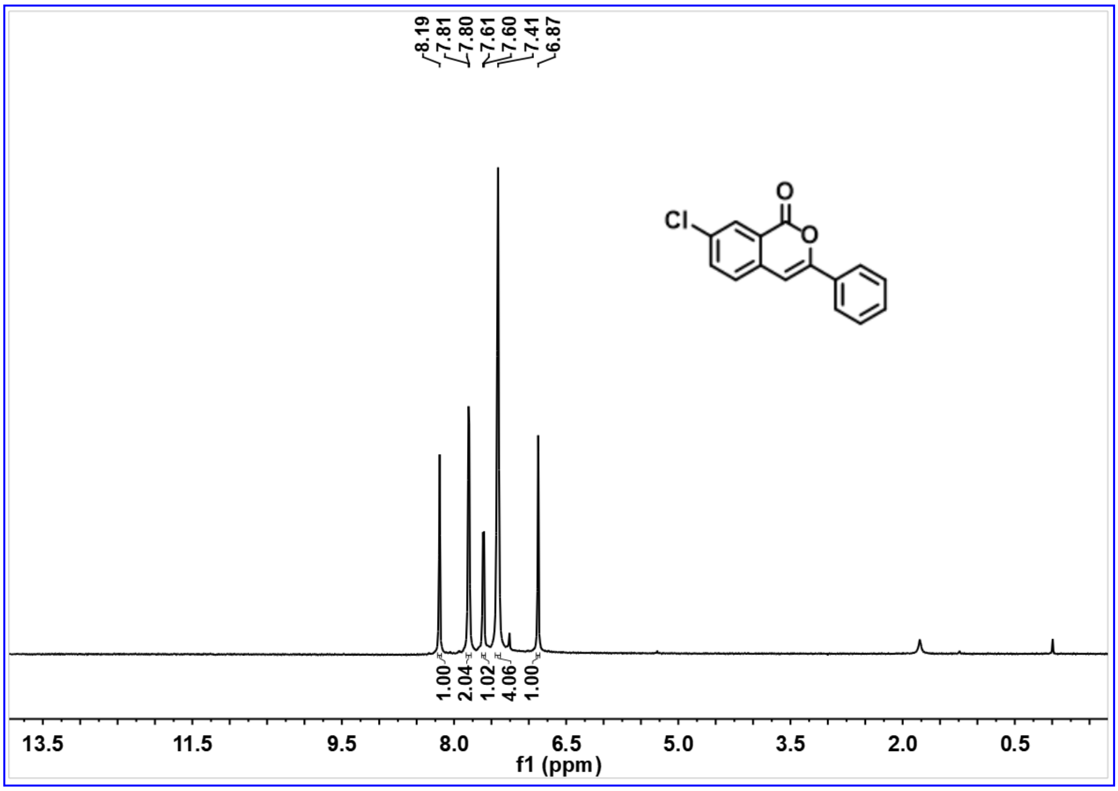

^{13}C{^{1}H} NMR (150 MHz, $CDCl_3$) of compound **4l**

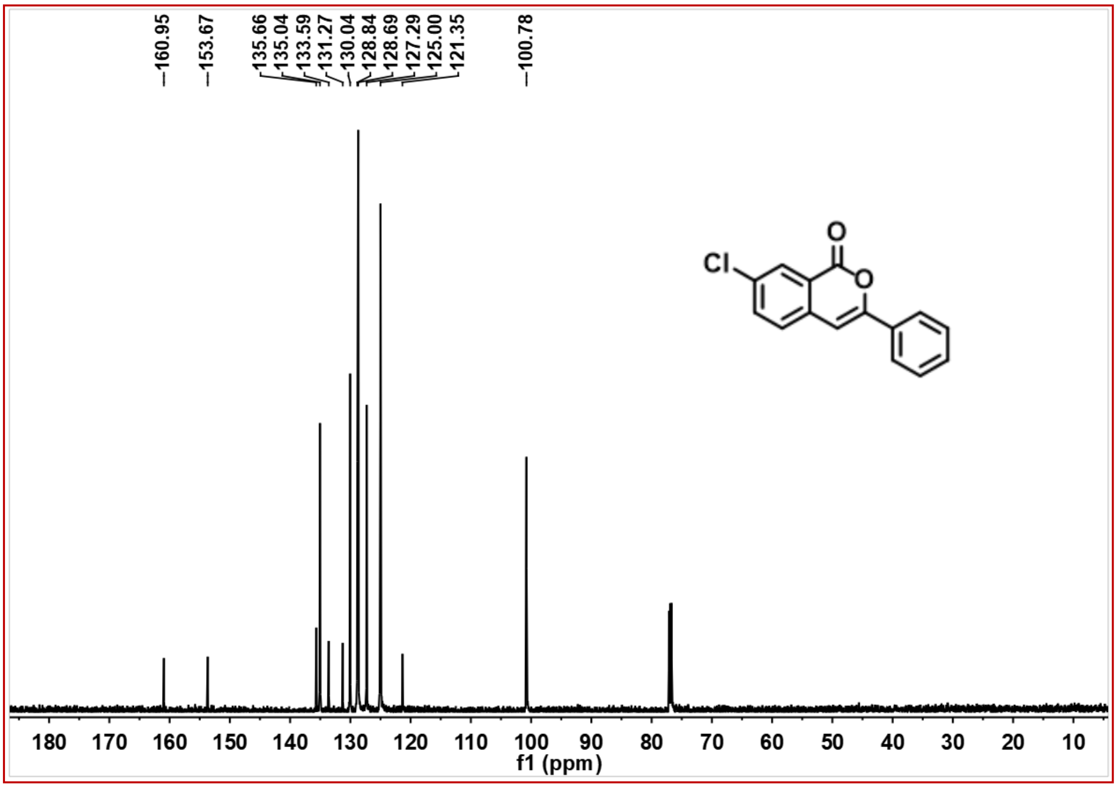

^{1}H NMR (600 MHz, $CDCl_3$) of compound **4m**

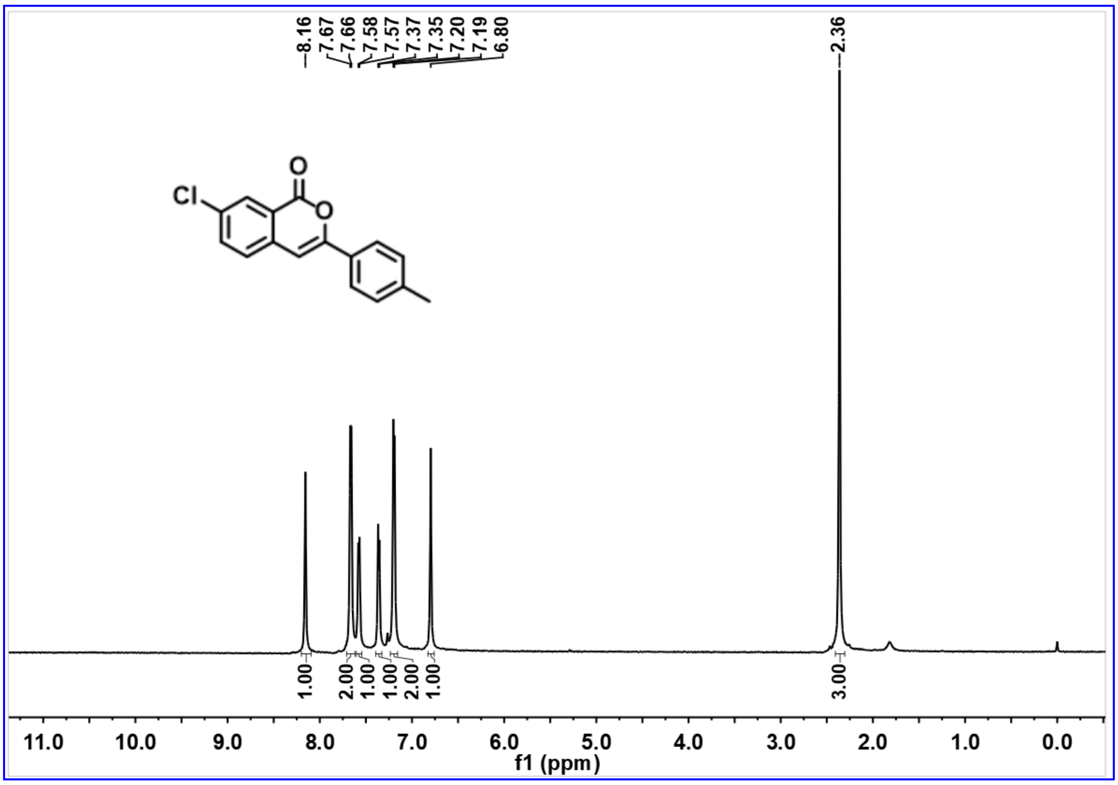

^{13}C{^{1}H} NMR (150 MHz, $CDCl_3$) of compound **4m**

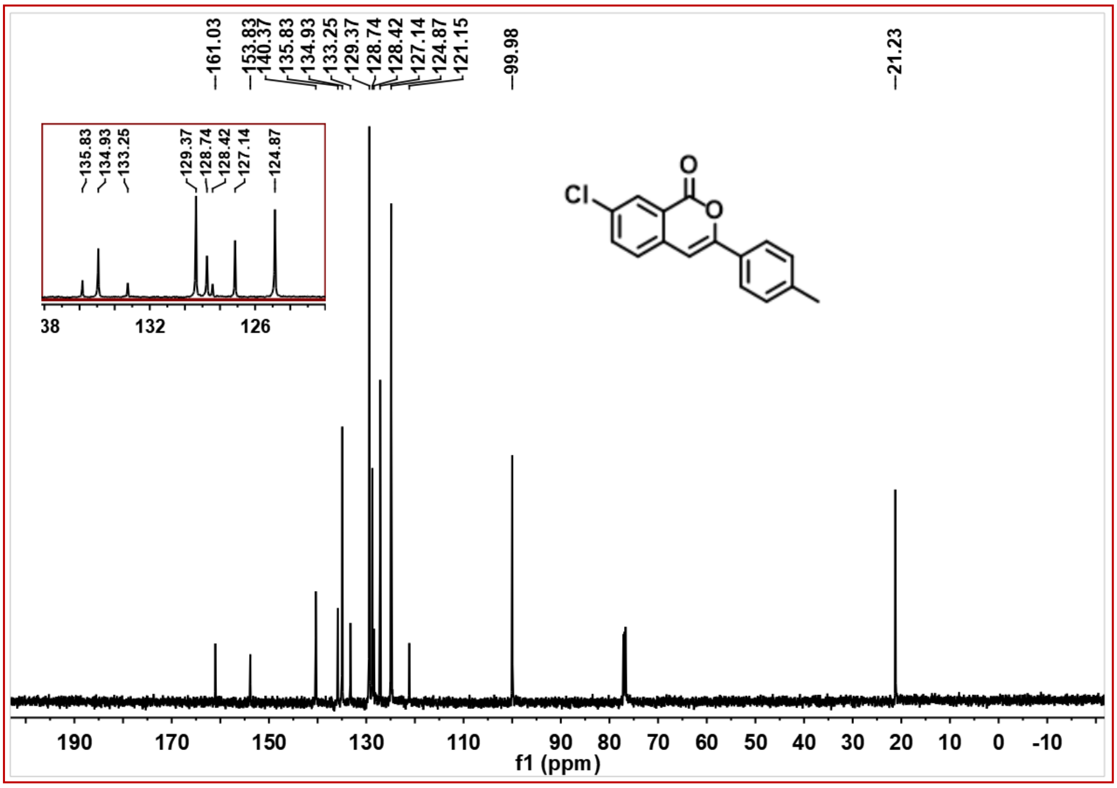

^{1}H NMR (600 MHz, $CDCl_3$) of compound **4n**

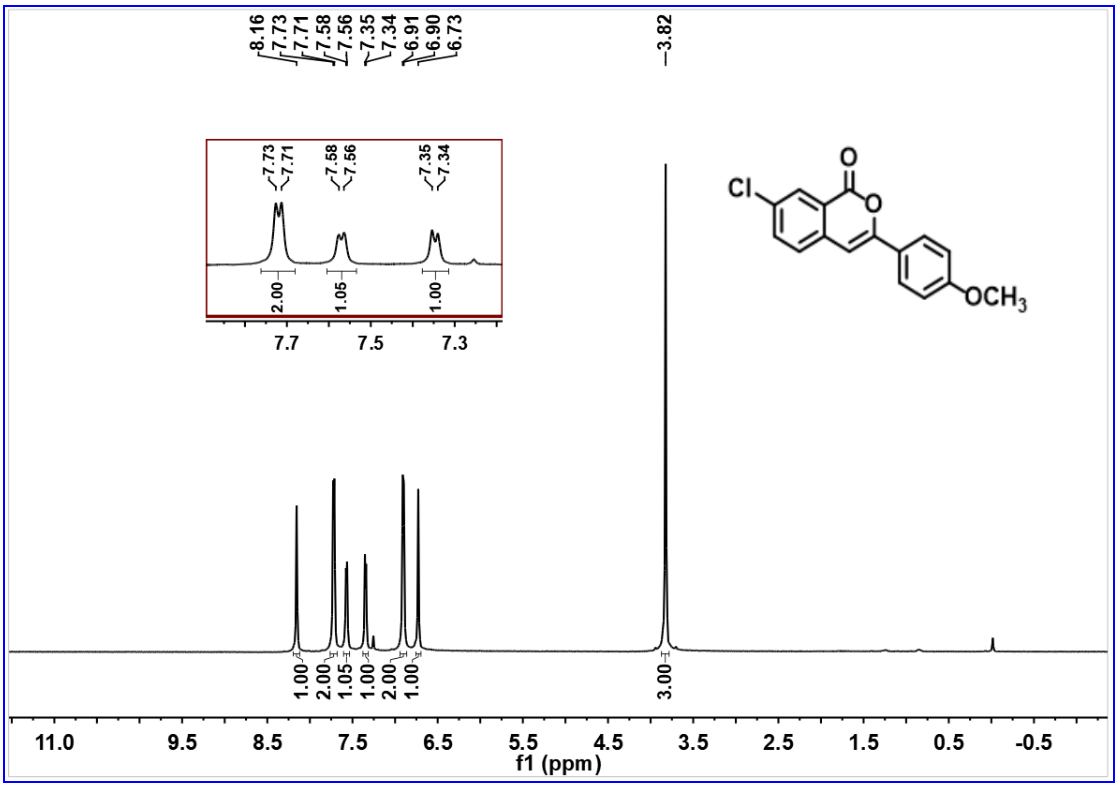

^{13}C{^{1}H} NMR (150 MHz, $CDCl_3$) of compound **4n**

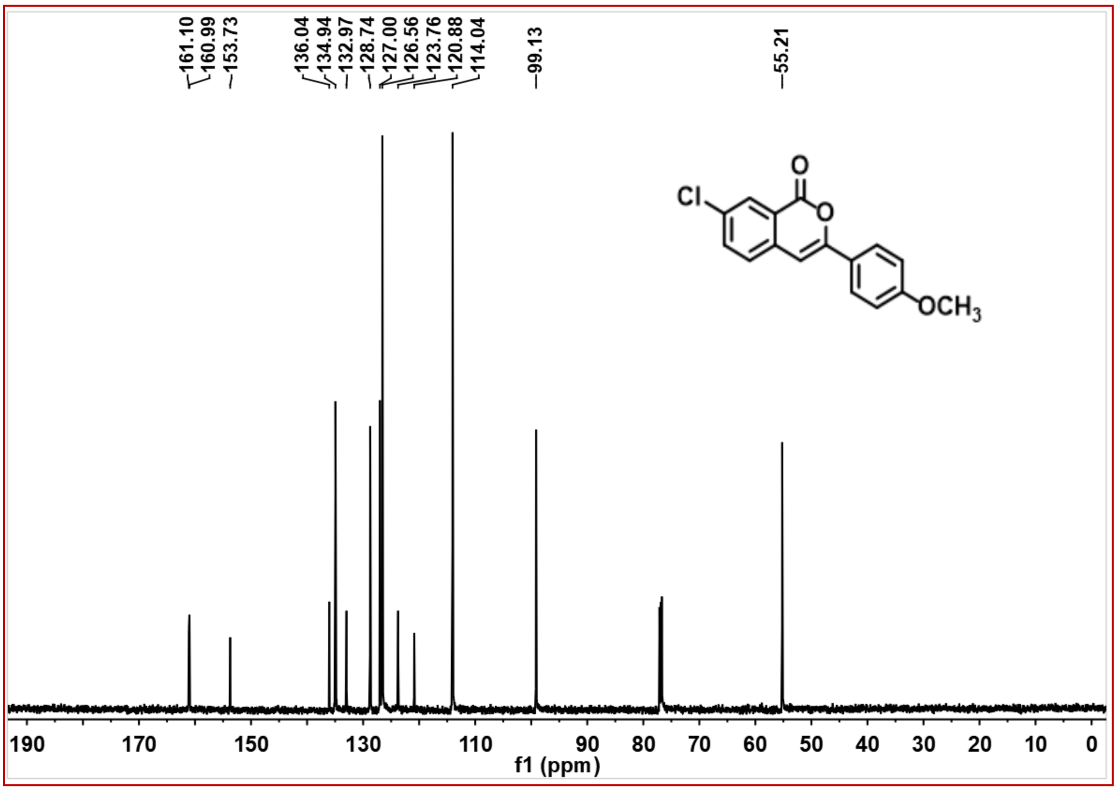

^{1}H NMR (600 MHz, $CDCl_3$) of compound **4o**

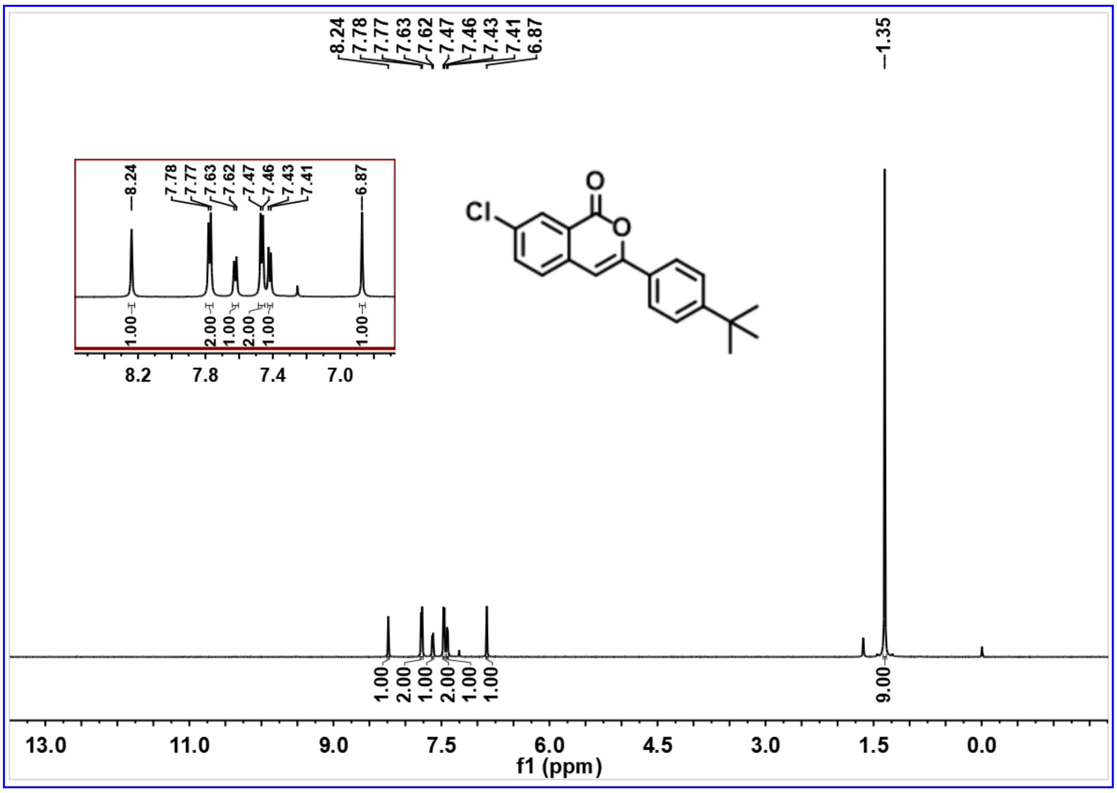

^{13}C{^{1}H} NMR (150 MHz, $CDCl_3$) of compound **4o**

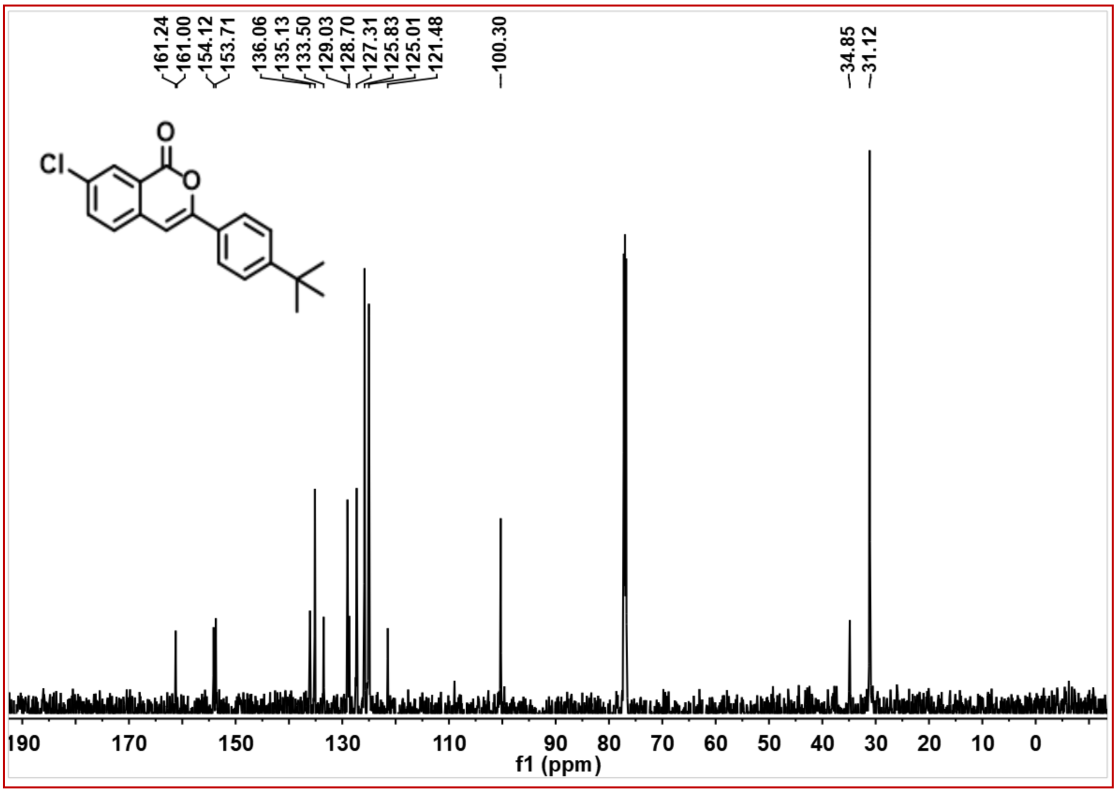

^{1}H NMR (600 MHz, $CDCl_3$) of compound **4p**

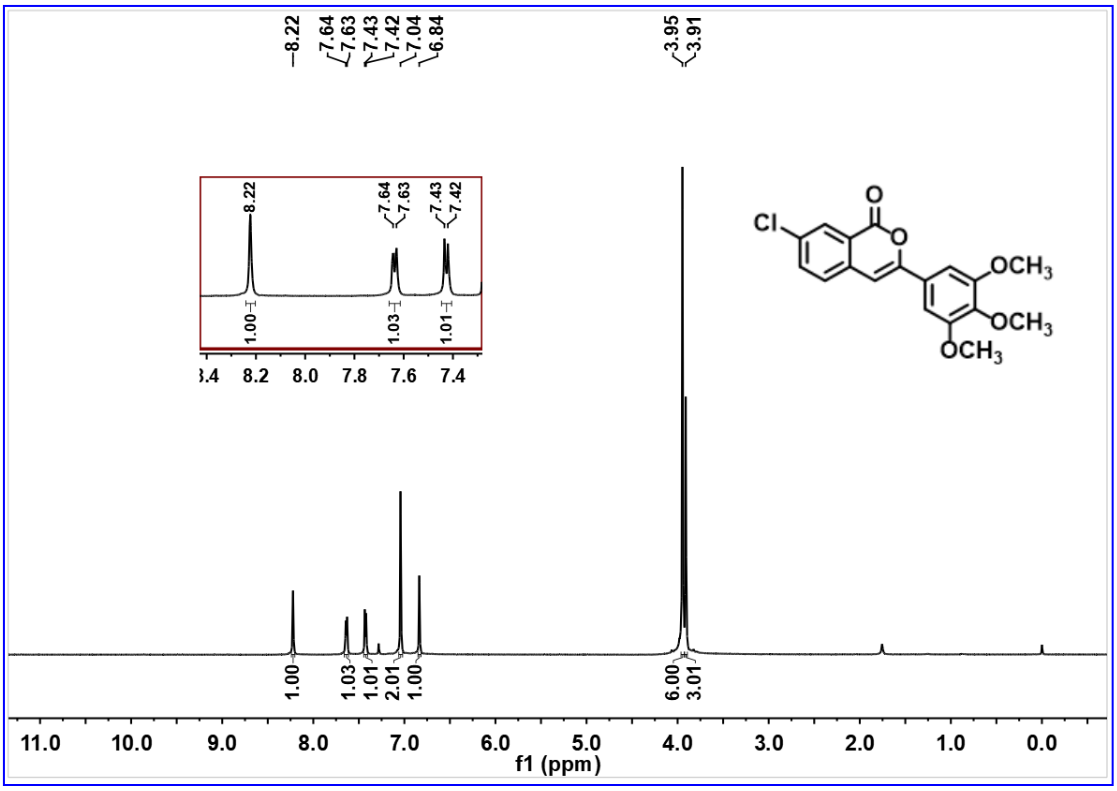

^{13}C{^{1}H} NMR (150 MHz, $CDCl_3$) of compound **4p**

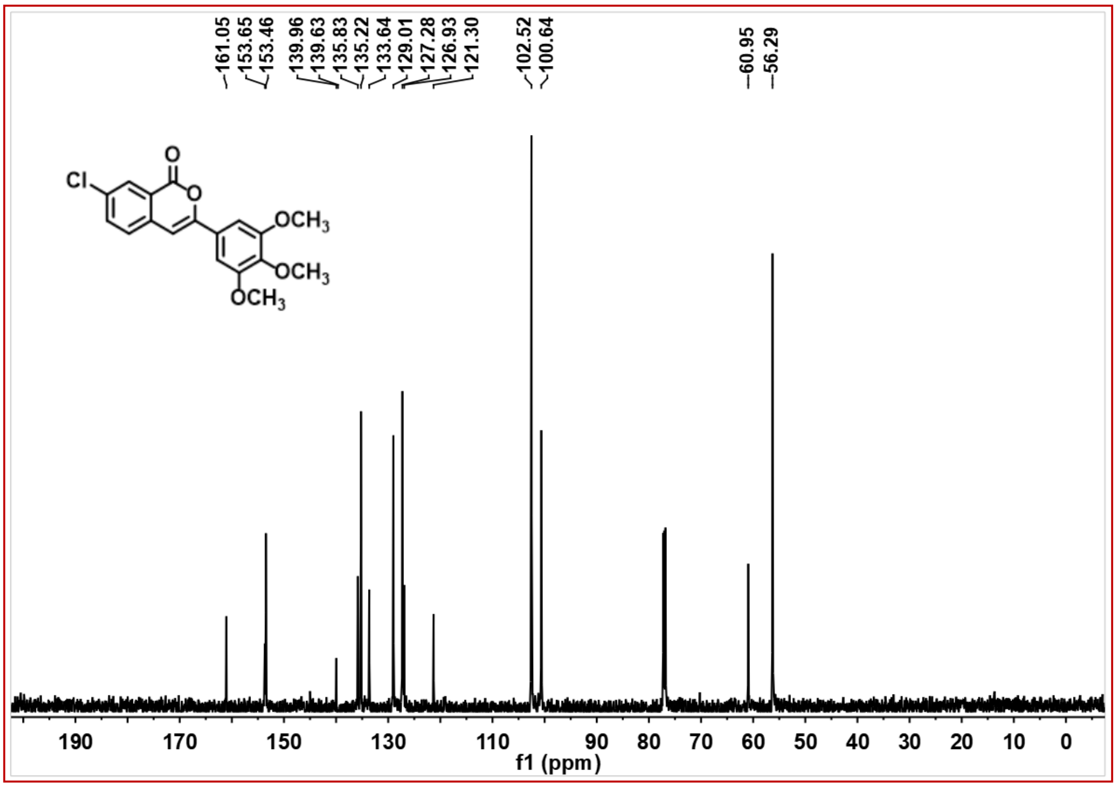

^{1}H NMR (600 MHz, $CDCl_3$) of compound **4q**

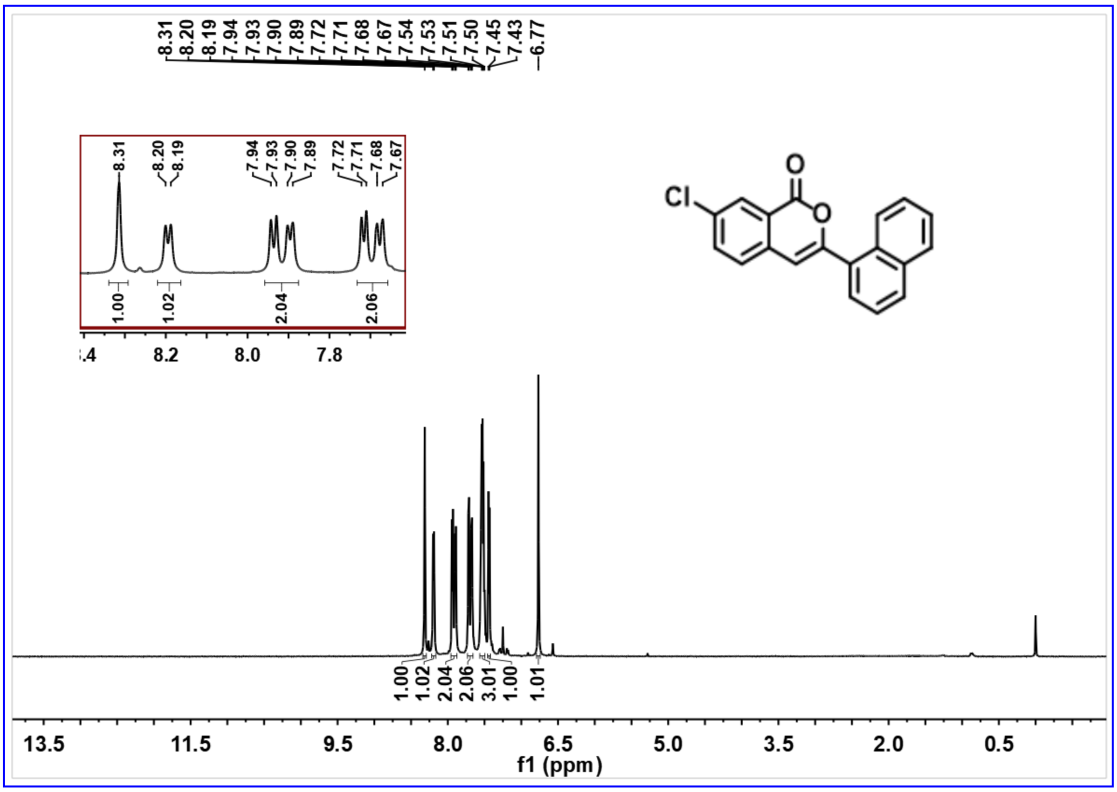

^{13}C{^{1}H} NMR (150 MHz, $CDCl_3$) of compound **4q**

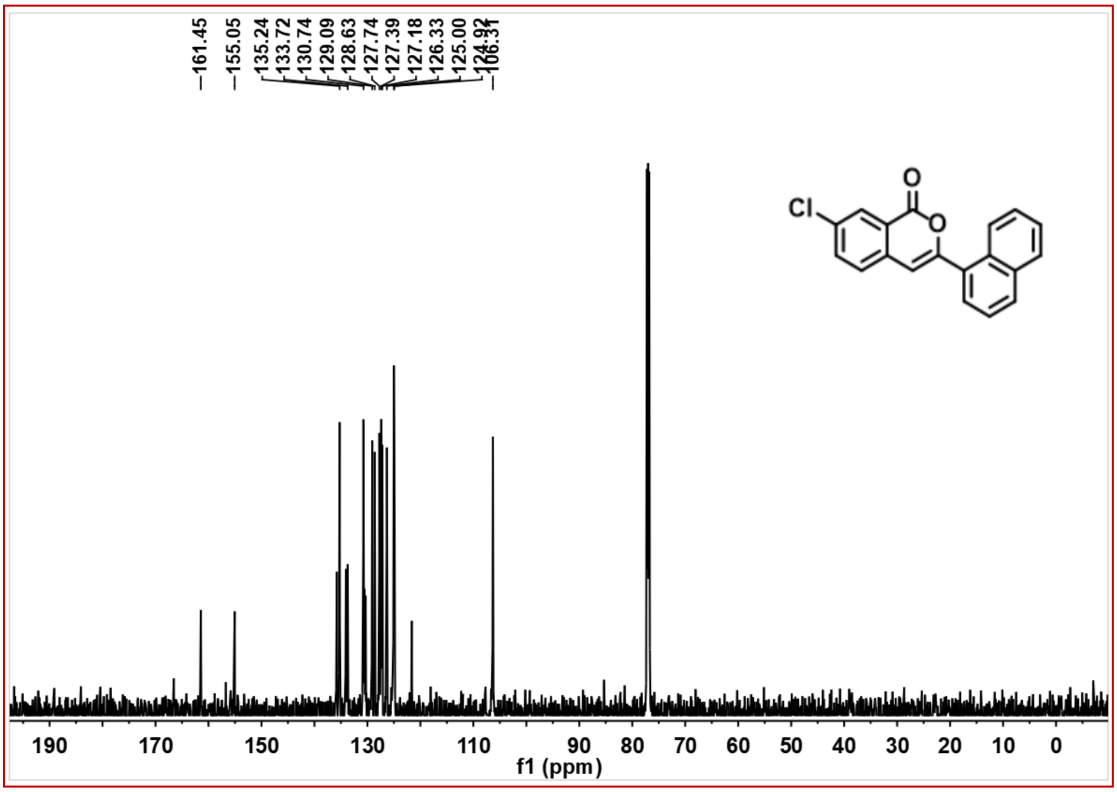

^{1}H NMR (600 MHz, $CDCl_3$) of compound **4r**

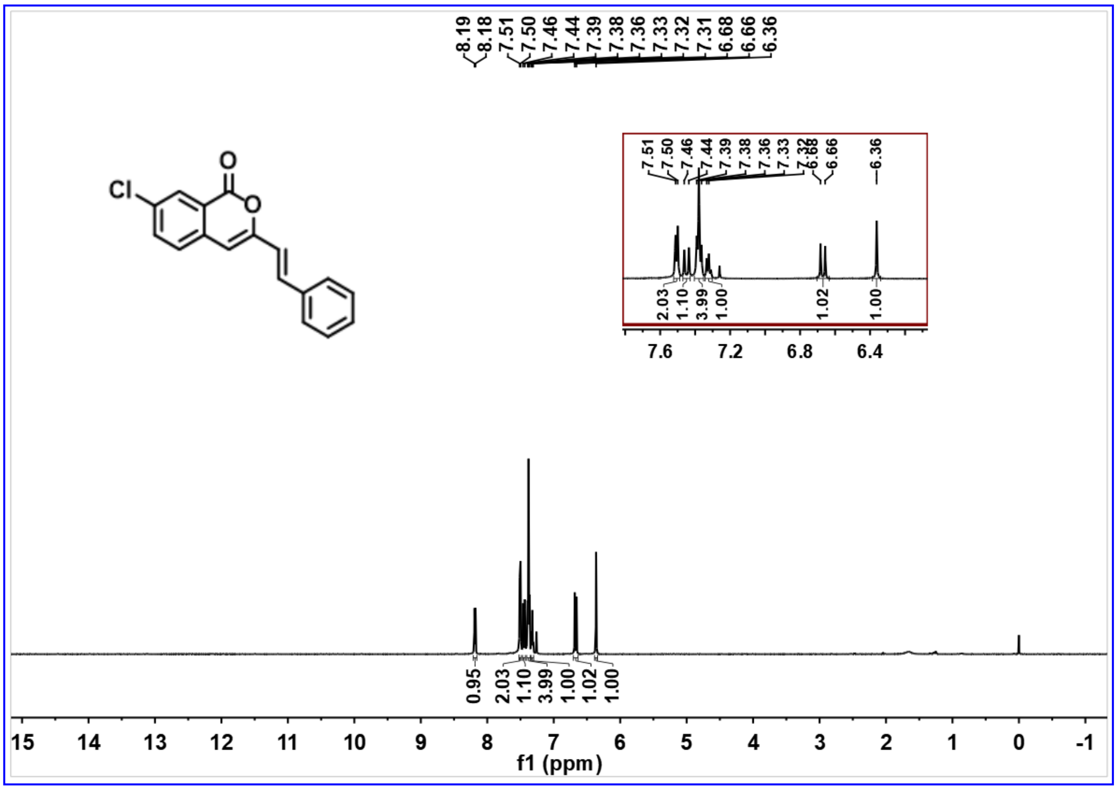

^{13}C{^{1}H} NMR (150 MHz, $CDCl_3$) of compound **4r**

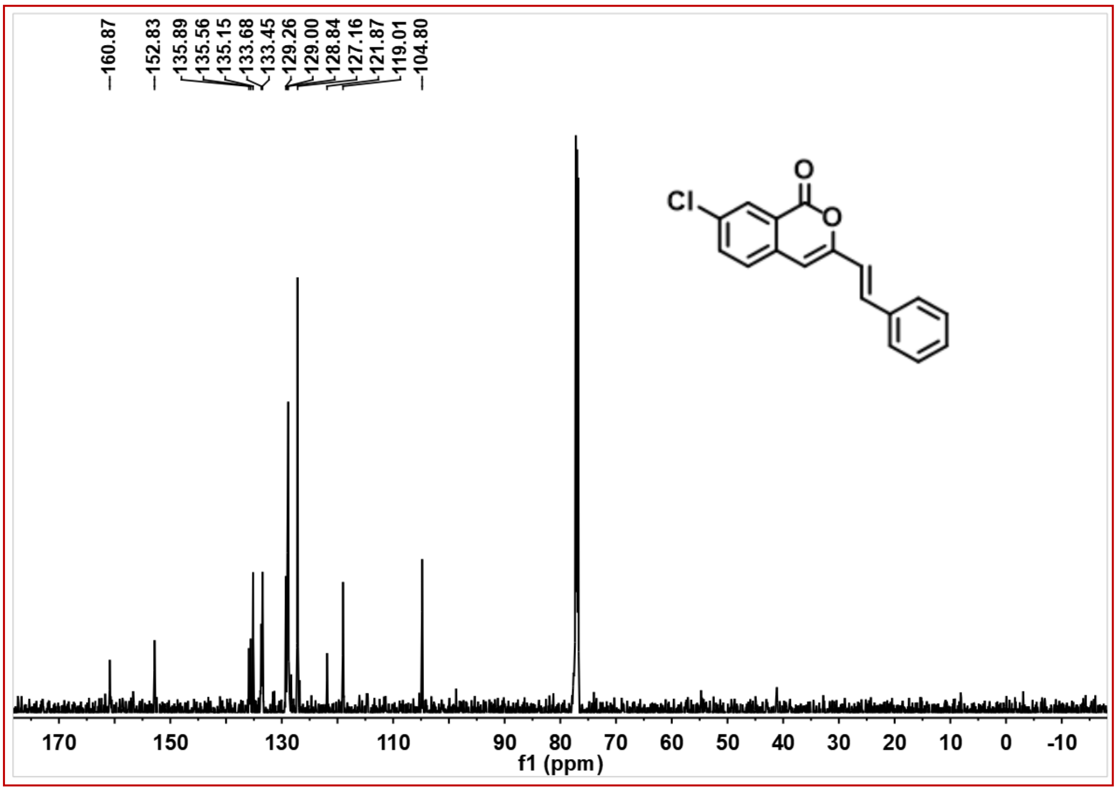

^{1}H NMR (600 MHz, $CDCl_3$) of compound **4s**

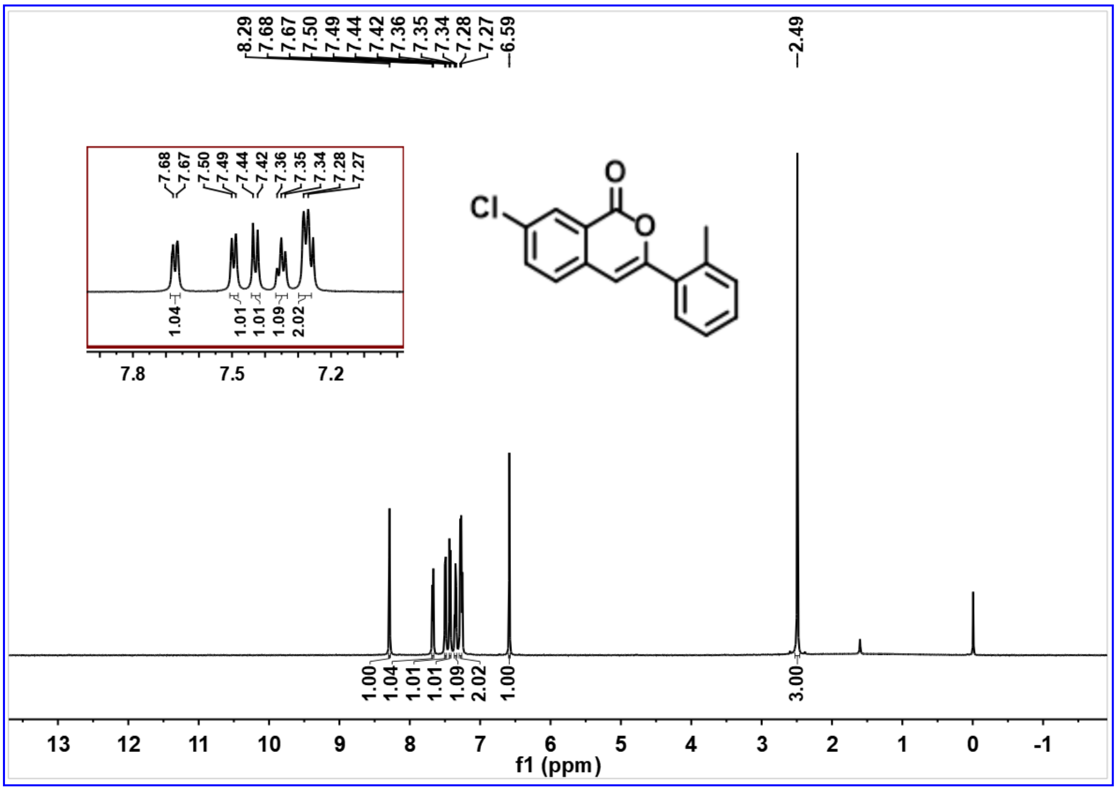

^{13}C{^{1}H} NMR (150 MHz, $CDCl_3$) of compound **4s**

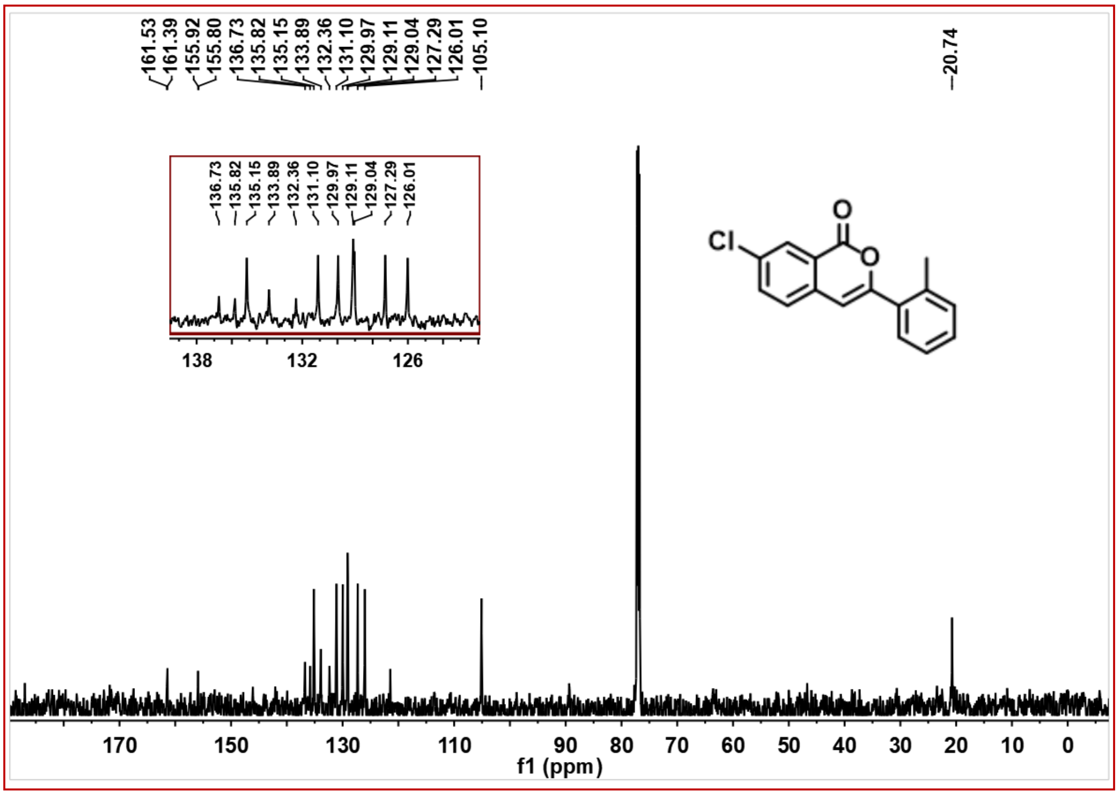

^{1}H NMR (600 MHz, $CDCl_3$) of compound **4t**

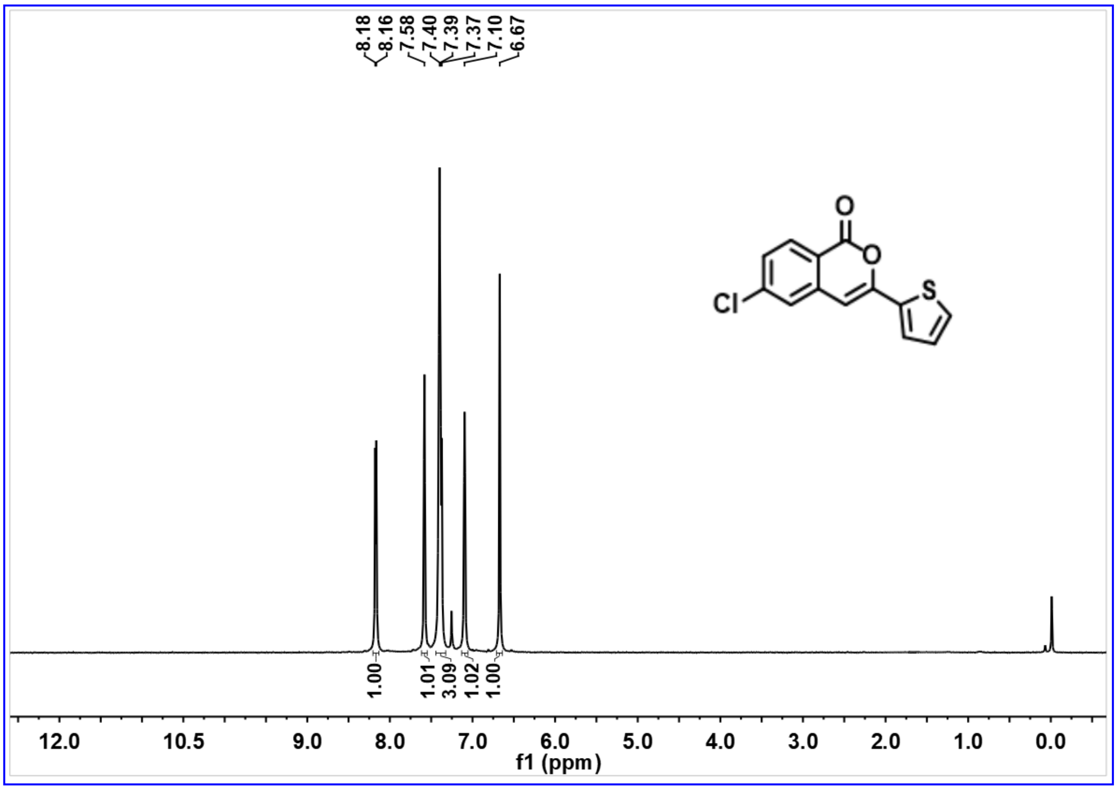

$^{13}C\{^{1}H\}$ NMR (150 MHz, $CDCl_3$) of compound **4t**

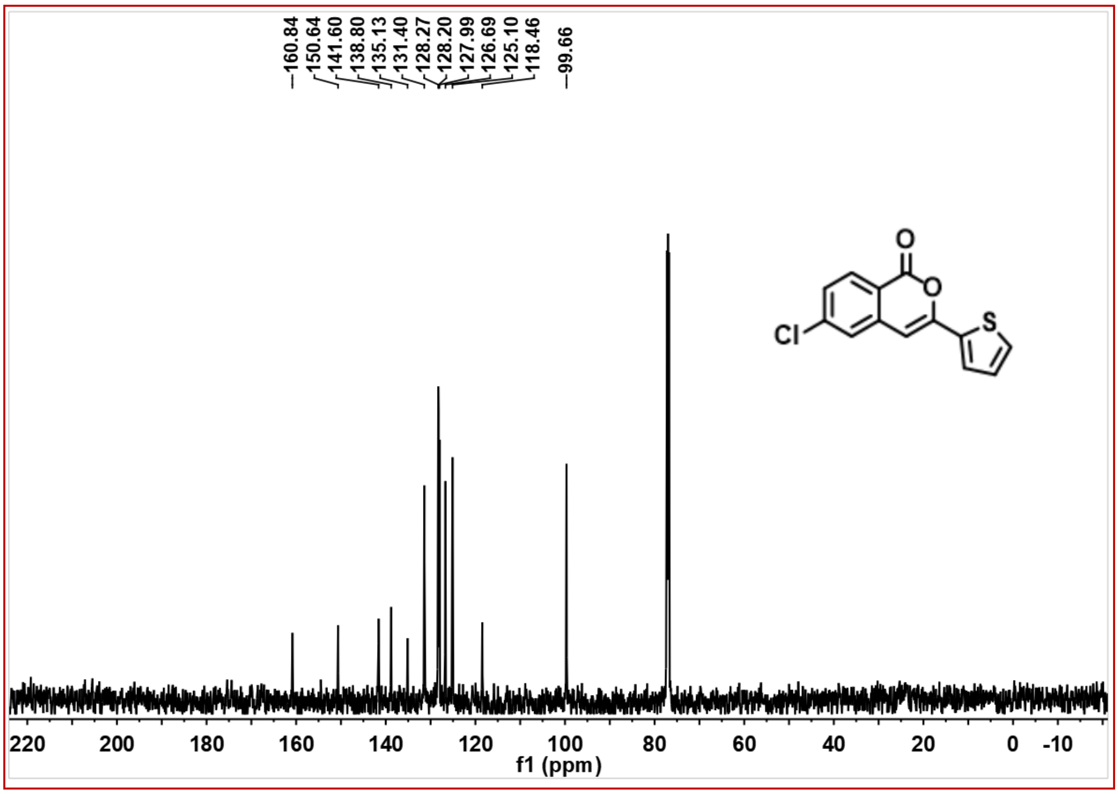

^{1}H NMR (600 MHz, $CDCl_3$) of compound **4u**

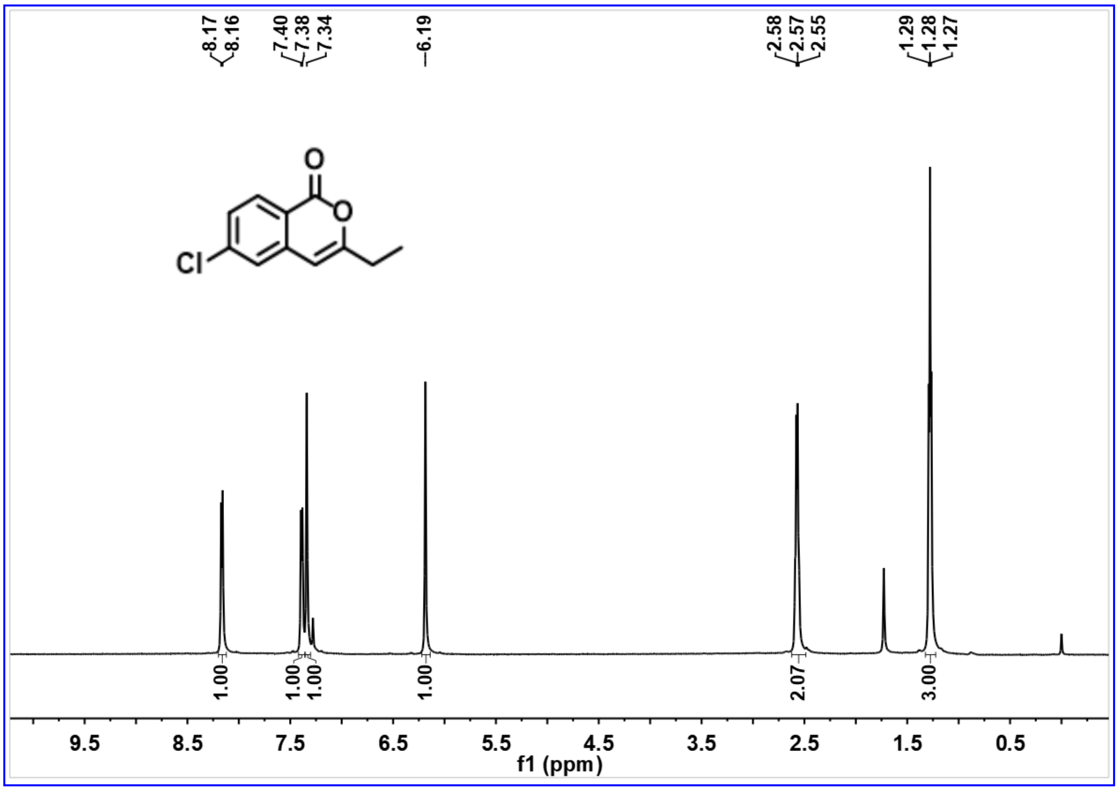

$^{13}C\{^1H\}$ NMR (600 MHz, $CDCl_3$) of compound **4u**

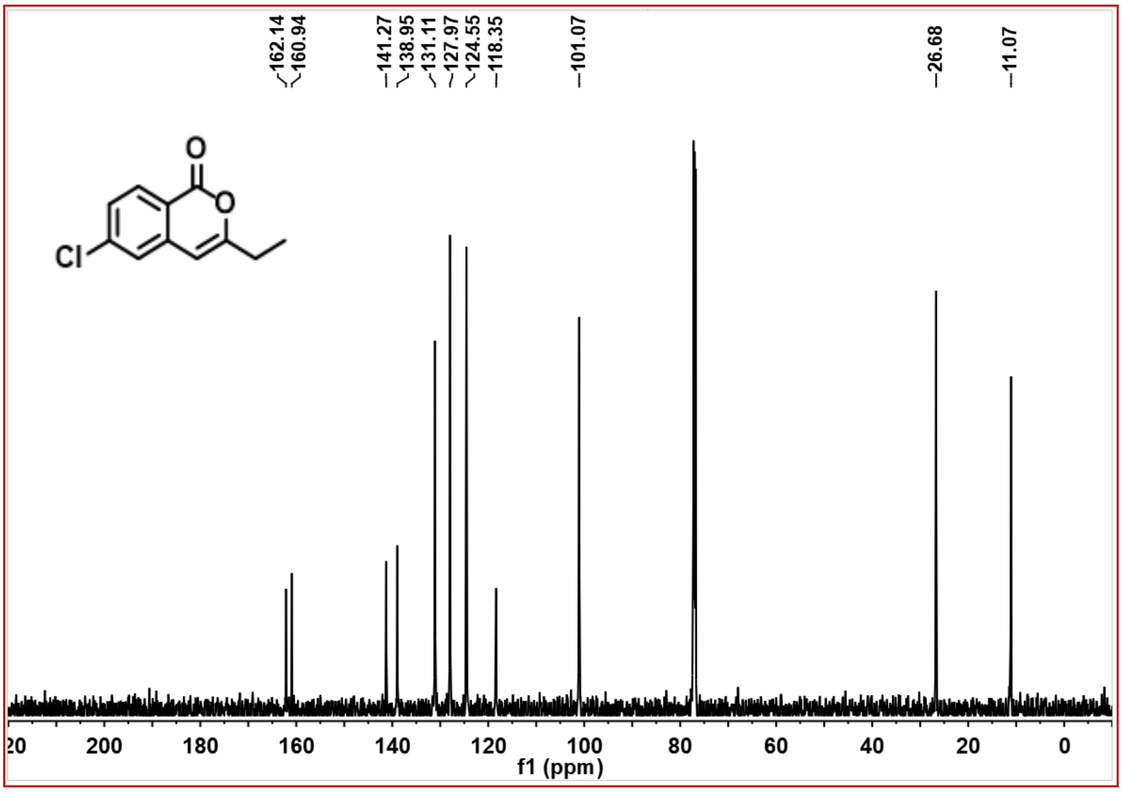

Supplement: Supplementary file 1 [file molecules-29-02449-s001.zip › molecules-2975328-supplementary.pdf]
